# Developing Subdomain Allocation Algorithms Based on Spatial and Communicational Constraints to Accelerate Dust Storm Simulation

**DOI:** 10.1371/journal.pone.0152250

**Published:** 2016-04-04

**Authors:** Zhipeng Gui, Manzhu Yu, Chaowei Yang, Yunfeng Jiang, Songqing Chen, Jizhe Xia, Qunying Huang, Kai Liu, Zhenlong Li, Mohammed Anowarul Hassan, Baoxuan Jin

**Affiliations:** 1 School of Remote Sensing and Information Engineering, Wuhan University, Wuhan, Hubei Province, China; 2 Center for Intelligent Spatial Computing, George Mason University, Fairfax, Virginia, United States of America; 3 Department of Computer Science, George Mason University, Fairfax, Virginia, United States of America; 4 Department of Geography, University of Wisconsin‑Madison, Madison, Wisconsin, United States of America; 5 Yunnan Provincial Geomatics Center, Kunming, Yunnan, China; Bangladesh University of Engineering and Technology, BANGLADESH

## Abstract

Dust storm has serious disastrous impacts on environment, human health, and assets. The developments and applications of dust storm models have contributed significantly to better understand and predict the distribution, intensity and structure of dust storms. However, dust storm simulation is a data and computing intensive process. To improve the computing performance, high performance computing has been widely adopted by dividing the entire study area into multiple subdomains and allocating each subdomain on different computing nodes in a parallel fashion. Inappropriate allocation may introduce imbalanced task loads and unnecessary communications among computing nodes. Therefore, allocation is a key factor that may impact the efficiency of parallel process. An allocation algorithm is expected to consider the computing cost and communication cost for each computing node to minimize total execution time and reduce overall communication cost for the entire simulation. This research introduces three algorithms to optimize the allocation by considering the spatial and communicational constraints: 1) an Integer Linear Programming (ILP) based algorithm from combinational optimization perspective; 2) a K-Means and Kernighan-Lin combined heuristic algorithm (K&K) integrating geometric and coordinate-free methods by merging local and global partitioning; 3) an automatic seeded region growing based geometric and local partitioning algorithm (ASRG). The performance and effectiveness of the three algorithms are compared based on different factors. Further, we adopt the K&K algorithm as the demonstrated algorithm for the experiment of dust model simulation with the non-hydrostatic mesoscale model (NMM-dust) and compared the performance with the MPI default sequential allocation. The results demonstrate that K&K method significantly improves the simulation performance with better subdomain allocation. This method can also be adopted for other relevant atmospheric and numerical modeling.

## Introduction

Numerical modeling has been an essential method for scientists and engineers to explore complex problems of physical dynamics. It enables predicting the course of an event before it actually occurs, or studying various aspects of an event mathematically without actually running expensive and time-consuming experiments. Using suitable and realistic numerical models, better understanding of practical problems can be obtained with relatively small effort [1]. The emergence of new computing technology further enables the exploration of more complex problems. For example, numerical modeling has been widely used in earth science, including atmosphere, geology, and oceanography.

Dust model is a typical numerical model and is developed to predict the spatiotemporal patterns, evolution, and the magnitude order of dust concentration, emissions and deposition for up to 3–7 days. With the advent and improvement of the prescribed dust source information, satellite retrievals, and ground observations, a new generation of specialized dust models have been developed to generate predictions with considerable accuracy. Dust models can be classified by their geographic coverage into regional or global coverage. Models with near global coverage, such as the Barcelona Supercomputing Centre-Dust Regional Atmospheric Model 8b v2.0 (BSC-DREAM8b) [2], are able to provide forecasts of the dust storm life cycle. As a regional model, Chinese Unified Atmospheric Chemistry Environment for Dust (CUACE/Dust) [3] model is an integral part of a real-time mesoscale sand and dust storm forecasting system for eastern Asia, and has an aerosol module that can differentiate the size of suspended particles. The model used in this study, NMM-Dust, is a meteorological core coupled with a dust module [4]. The meteorological core is the non-hydrostatic mesoscale model (NMM), which is also used in US National Weather Service (NWS) operations. NMM-Dust can produce dust load and dust concentration in up to 3km spatial resolution.

**Fig 1:**
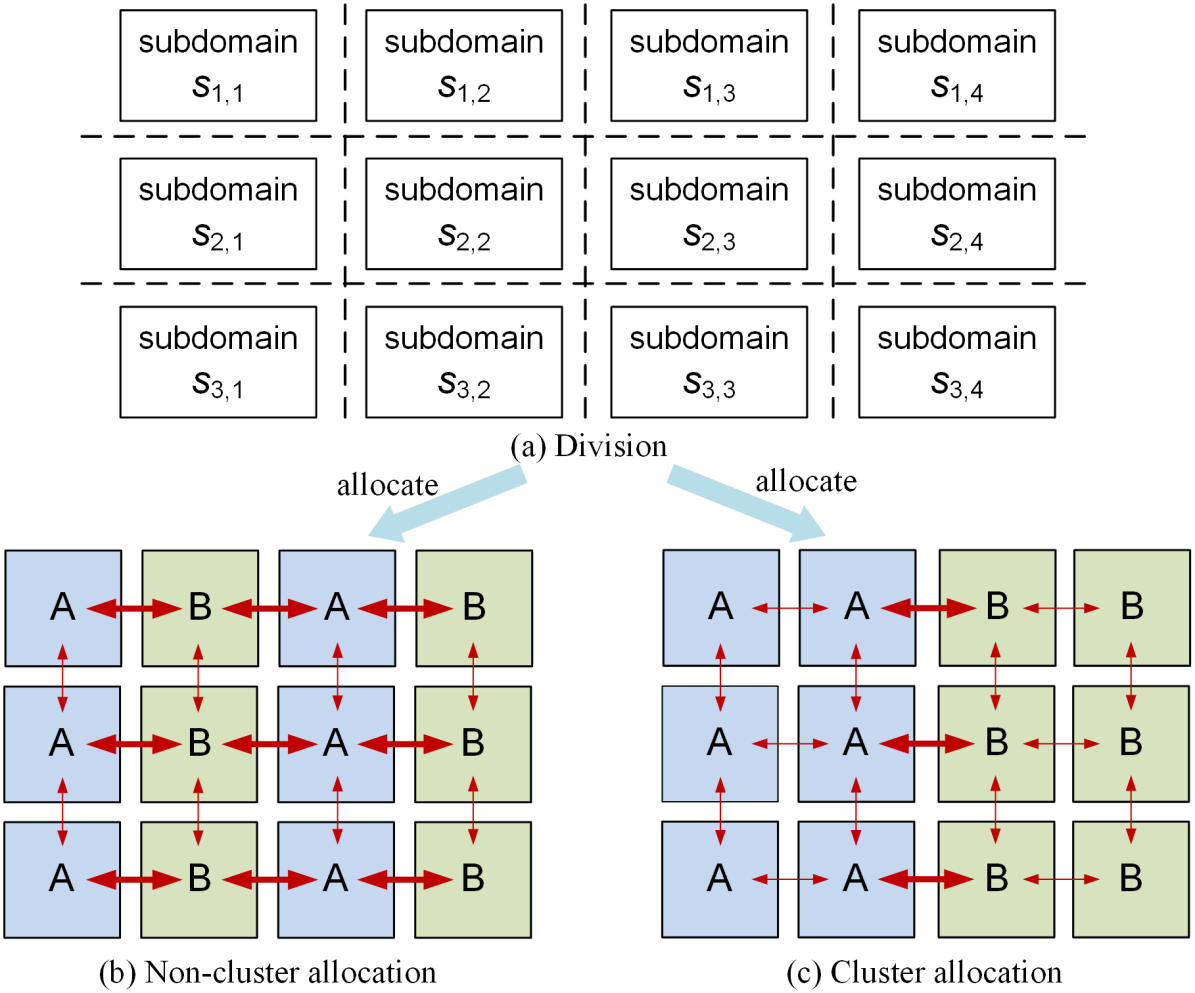
Parallelization of Dust Model (Domain Division and Two Allocation Methods for Dispatching 12 Subdomains to Two Computing Nodes, A and B).

However, dust modeling is highly computing intensive due to repetitive numerical calculations, vast dataset manipulations, and dust’s intrinsic four-dimensional feature (a time dimension, two horizontal dimensions, latitude and longitude, and one vertical dimension) [4–7]. In order to accelerate the simulation, parallelization is adopted by using message passing interface (MPI) programming model [8]. Firstly, a Single Program Multiple Data (SPMD) decomposition approach is used to decompose the domain evenly into multiple or many subdomains, which are then allocated to multiple computing nodes. The two-step process is called domain decomposition. Fig 1 illustrates the decomposition process for a single vertical layer. Since spatiotemporal correlations exist in the geophysical process of dust storm simulation, each subdomain needs to communicate with other geographically adjacent subdomains to exchange data for local computation and synchronization frequently [6]. Red arrows in Fig 1 demonstrate the communication between subdomains. There are two types of communications (external and internal). When two neighboring subdomains are allocated on two computing nodes separately, the intermediate data result of a subdomain generated on one computing node is transferred to another node across computer network. It inevitably introduces external communication. In the meantime, internal communication happens with data exchanged among simulation subdomains within the same single computing node. Comparing with external communication which introduces network I/O, internal communication cost (i.e., local disk/in-memory I/O) is trivial and can be ignored to some extent. In this paper, communication refers to external communication, except for some specified occasions.

The cost of data transfer due to communication among neighbor subdomains is a key efficiency issue because it adds significant overhead [9]. Different subdomain allocation methods result in different communication overheads among the subdomains [6]. Fig 1(b) and 1(c) show two types of allocation methods for allocating 12 subdomains to two computing nodes A and B. Since MPI is not responsible for scheduling, how subdomains are allocated to the computing nodes are customized by model developers and engineers. If there is no customized allocation, the system dispatches the subdomains to the computing nodes sequentially row after row (i.e., typical non-cluster allocation method as shown in Fig 1(b)). Fig 1(c) requires only 6 one way external communications over two different computing nodes while Fig 1(b) requires 18 external communications to exchange data over two computing nodes. Hypothetically, the method in Fig 1(c) can reduce the total communication overhead by making more communication occur within the same node rather than over computer networks. By using cluster allocation and non-cluster allocation methods to run the same set of model simulation tasks under different decomposition granularities, Huang et al. [6] validated this hypothesis and indicated that cluster allocation method achieved an average 20% performance improvement.

When dividing rules and the number of subdomains are specified for a given domain, the allocation method becomes a key issue to the simulation performance. Therefore, it is desirable to find an optimized case-dependent subdomain allocation method. An optimized allocation requires to best leverage the computing capacity gains and communication costs for minimizing numerical calculation time. The dynamic and heterogeneous features of environments as well as spatial and communicational constraints should be considered [10]. A similar problem exists in Very-large-scale integration (VLSI) design and is treated as a typical Graph Partitioning Problem (GPP) [11, 12]. In the situation of GPP, the subdomains are represented as regularly meshed vertices, and the communications between neighboring subdomains are represented as the connection between vertices (4-neighbor or 8-neighbor). Specifically, a connection is depicted as a *shared edge* between subdomains in 4-neighbor situation. A partition of domain refers to an allocation of subdomains on multiple computing nodes. Each component (comprised of all subdomains allocated to the same computing node) with a group of vertices (corresponding to the subdomains) is allocated to a single computing node. Dust storm simulation utilizing high performance computing requires subdomain allocation in multiple computing nodes, so that it can also be an application benefited from GPP. Besides minimizing total shared edges, the amount of vertices (i.e., subdomains) as well as the (weighed) shared edges of each component needs to be balanced. As an active research area, many categories of GPP algorithms have been developed in the past decades. It is desirable to leverage the most suitable GPP algorithms with acceptable performance to better support dust storm simulation. This paper designs and compares three subdomain allocation algorithms from different categories of GPP (i.e., combinatorial optimization, global clustering combined with local heuristic, and raster-based local geometric partitioning). The proposed algorithms can be applied to other numerical simulation applications that require domain decomposition. The remainder of this paper is organized as follows: Section 2 reviews related work on GPP which are targeted at domain decomposition. Section 3 introduces the proposed algorithms. Section 4 reports the experiment results. Finally, Section 5 concludes the paper and discusses future research.

## Literature Review

Domain decomposition plays a vital role in high-performance scientific modeling and numerical simulations, such as dust modeling [13–16]. It enables parallel computing by dividing large computational task (i.e., data set) into small pieces and allocating them onto different computing resources (as illustrated in Fig 1). In order to efficiently execute the numerical simulation in a homogenous parallel computing environment (e.g., a high performance cluster), graph partitioning algorithms can be used to optimize the allocation process by balancing the workload as well as minimizing the inter-node or inter-processor communication, which performs data exchange between adjacent subdomains [17–19]. However, partitioning a graph into balanced sub-graphs with minimum connection is a NP-Complete problem [20, 21] and currently there is no efficient method that gives optimal partitioning in polynomial times. Solutions to these problems are generally derived using heuristic and approximation algorithms. Algorithms based on heuristics can be categorized from different perspectives, e.g., local and global. In this section, we review several categories of graph partitioning algorithms, which target on high performance scientific simulation, from the perspectives of basic algorithms techniques and describe the motivation for proposing our algorithms.

### Combinatorial Methods and Combinatorial Optimization

Combinatorial methods classify vertices with high connecting relations to them regardless of whether or not they are near each other in space [19]. Kernighan-Lin algorithm [22] is one of the earliest and most well-known local heuristic algorithms. It adopts bisectional method, which takes two separate components as an initial solution (i.e., two-way cuts), and exchanges pairs of vertices between them in order to reduce connections between two components (local search strategy). The algorithm has important applications in the layout of digital circuits and components in VLSI [23]. The major drawback is that the arbitrary initial partitioning of the vertex set may affect the final solution quality. Furthermore, the algorithm has high time complexity (O((m*n)^2^)log(m*n))), so that solving large partitioning problem might be exclusively time-consuming. A number of iterative refinement algorithms have been developed to target graphs of a specific topology, such as the cyclic pairwise exchange algorithm of Hammond [24], and the 1D or 2D grids partitioning by Walshaw et al. [25]. As a refinement of Kernighan-Lin, Fiduccia-Mattheyses algorithm moves only a single vertex between components instead of swapping pairs of vertices [26]. In most cases of these algorithms, the iterative migration of the vertices occurs by using a modified version of Kernighan-Lin algorithm.

On the other hand, non-specific direct methods are either numerical methods such as linear programming and quadratic assignment [27, 28], or metaheuristics [29–31]. Linear programming (LP) based algorithms provide better cuts than Kernighan-Lin. Leighton and Rao [32] used linear programs to study the maximum flow and the minimum cut in multi-commodity flow problems. Khandekar et al. [33] built upon expander flows to present a new approach for discovering graph separators that use single commodity flow. Besides, Lisser and Rendl [34] used linear and semi-definite programming to solve graph partitioning in the context of the telecommunication networks. However, the shortcoming of LP based algorithms lies in the performance of handling large partitioning problems, resulting in inability to complete in real or near real time manner.

Metaheuristics has been adapted to graph partitioning optimization problems. Among the adaptions, local search metaheuristics methods are greatly applied, including simulated annealing [30, 31, 35], GRASP (Greedy Randomized Adaptive Search Procedure) [36], tabu search [37, 38], and variable neighborhood search (VNS) [39]. These algorithms use heuristics to proceed from one solution to another, and/or refine these solution. Although they have good adaptability to different objective functions and constraints of graph partitioning optimization problems, these adaptations are quite time-consuming, compared to multilevel methods. The solutions developed by these adaptions are of the same quality as those found by Kernighan-Lin algorithm, but are clearly worse than those of multilevel method. However, these adaptations are found to obtain better results and high efficiency used in conjunction with a multilevel method [40, 41].

### Geometric Methods

Geometric techniques calculate partitions based on the coordinate information of the vertices [42, 43] and attempt to group vertices that spatially near each other, while the connectivity of vertices is not considered. Recursive Bi-partitioning [44], Space Filing Curve Techniques [45] and divide-and-conquer approximation algorithms [46] are well-known methods.

Clustering methods aim to reduce the complexity of the partitioning problem, by decoupling the source graph partitioning problem and the problem of allocating the obtained parts on the target graph. The clustering of vertices of the source graph can be done in two ways: either downward, by partitioning the source graph in as many parts as the vertices in the target graph, such as the recursive bi-partitioning [47], or upward, by hierarchical aggregation of vertices connected by edges of heaviest weight [48]. K-Means clustering [49] is a classical and widely-used cluster analysis method. The result of K-Means can be seen as the Voronoi cells of cluster means. Since each observation belongs to the cluster with the nearest mean, the Voronoi cells have relative short perimeters as well as total perimeters. Therefore, the Centroidal Voronoi Tessellations (CVT) generated by K-Means can balance the external communication of computing nodes to some extent [50]. Algorithms derived from K-Means have been applied in graph partitioning and data partitioning [51–53]. Yan and Hsiao [54] presented a fuzzy clustering algorithm to solve the graph bisection problem and apply to circuit partitioning. However, the observation number in one cluster of the K-Means solution may deviate a lot from one to another, so that the task load might not be well balanced. Additionally, the clustering result of K-Means depends heavily on the selection of initial centroid points. Different initial points can generate different results, even resulting in empty clusters.

Space fill curve technique is a fast partitioning method which fills up high dimensional space (e.g., square or cube) with continuous curves in a locality-preserving fashion [19, 45]. Adams and Bischof [55] presented a Seeded Region Growing (SRG) algorithm for gray-color image segmentation. SRG is an efficient, robust and intuitive algorithm, but it requires several pre-prepared control points as original seeds. Shih and Cheng [56] proposed an optimized automatic SRG algorithm used for color image segmentation. Shan et al., [57] provided a completely automatic SRG to segment ultrasound images by selecting seed points based on textural and spatial information. Sphere-cutting approaches use the concept *overlap graphs* to conduct partitioning for well-shaped meshes (i.e., the meshes in which the angle or the aspect ratios are strictly bounded) [19, 58].

### Evolutionary Algorithms, Swarm Intelligence and Combined Methods

Evolutionary and swarm intelligence approaches are also proposed, such as genetic algorithms [59–62] and ant colony optimization [63, 64]. Genetic algorithms are search algorithms based on the principle of natural selection and genetics [61, 62]. The well-known advantages of GAs are that they tend to find global or strong local optima, operate without any derivative information, work for functions that depend on both discrete and continuous variables, and are simple to create and maintain [61]. However, GAs have typically been applied only to those synthesis problems where the corresponding analysis is computationally cheap. They require too many function evaluations for application to many problems of interest. Similarly, ant colony optimization uses a metaheuristic approach for solving hard combinatorial optimization problems [63]. It is based on the indirect communication of a colony of simple agents, called (artificial) ants, mediated by (artificial) pheromone trails. Ant colony optimization algorithm performs very well on small or medium-sized graphs, while with larger graphs, which are encountered in our partitioning problem, it has to be combined with a multilevel [65] or other improving mechanisms.

Since all algorithms have their unique advantages and disadvantages, a combined method can maximize the advantages and reduce the disadvantages if designed appropriately. Initial solutions used as starting points by iterative algorithms are obtained using greedy partitioning methods of low cost, such as region growing method in [66] or a breadth-first search in Gibbs-Poole-Stockmeyer algorithm [67]. These algorithms aim to provide an initial solution consisting of balanced and predominantly compact and connected partitions, where the iterative algorithms seek to improve the cost function, while maintaining the balance. Multilevel methods, the most commonly explored methods in these days, can be seen as an optimization of the clustering methods [68] by utilizing combined scheme. The method is divided into three successive and well-distinct levels: Coarsening, Partitioning, and Uncoarsening and refinement. In many cases, multilevel approach can provide both fast execution times and high quality solutions (as good as linear programming based methods). Karypis and Kumar [69, 70] presented a widely used graph partitioner METIS and hypergraph partitioner hMETIS [71]. Portugal and Rocha [72] proposed a k-balanced graph partitioning method based on the generalization of hierarchical multilevel bi-sectioning approach. This approach can partition undirected weighted graphs into any arbitrary number *k* of regions in a balanced way, up to the point where the graph can no longer be partitioned. However in this approach, the components share at least one vertex with neighboring components. Since the components are not totally disjoint, it is slightly different from our partitioning target, where all grids are separated and one grid cannot be in two different partitions. Similarly, Van Den Bout and Miller [73] described a partitioning algorithm that combines characteristics of the simulated annealing algorithm and the Hopfield neural network.

Most work cited herein attempt to partition graphs into two or more components while minimizing the sum of the (weighed) shared edges which have endpoints in different components. In general, combinatorial methods can achieve relatively good partitioning quality but have expensive computing cost [19, 22], so they may not be suitable for large source graphs for processing mapping applications. Geometric methods are efficient but have relatively poor partitioning quality compared with combinatorial methods [19]. Evolutionary algorithms are effective and capable to overcome local minima [60], but they are complicated and hard to be modelled due to the variety and complexity of parameters [61], e.g., the selection of initial population needs to guarantee or at least consider the diversity of population. Outperforming the previous methods, combined methods can utilize the advantages of distinct methods and try to avoid their disadvantages to some extent [69]. In the dust storm simulation, we explore graph partitioning algorithms that are viable to meet or best leverage the allocation requirements while achieving time-efficiency. Since different categories of GPP algorithms have their unique characteristics, three algorithms selected from three major categories (geometric, combinatorial and combined) respectively are introduced and compared. Their capabilities and application scenarios for a dust storm simulation are discussed.

1. An Integer Linear Programming (ILP) based algorithm designed from the perspective of combinatorial optimization as a global partitioning method. ILP is a widely-used operational research method which can consider the entire graph in partitioning, unlike other combinatorial methods (e.g., Kernighan-Lin and Fiduccia-Mattheyses) which only conduct localized refinement. If designed appropriately, ILP model can obtain optimal partitioning solution, but it will be costly when the problem size becomes large. Therefore, we use LP as a benchmark for comparison.
2. A K-Means and Kernighan-Lin combined heuristic algorithm (K&K) integrating clustering method and merging local and global partitioning together. K&K is a geometric and combinatorial combined method which generates initial partitioning using a standard K-Means and then refines partitioning using a modified Kernighan-Lin. Due to the global clustering capability of K-Means, K&K can maintain global shape to some extent while conducting localized refinement. Unlike multilevel method which contains the coarsening and uncoarsening procedures, K&K is conceptually simple but hasn’t been investigated.
3. An automatic-seeded-region-growing-based geometric and local partitioning algorithm (ASRG) inspired by the application of graph partitioning to image segmentation. ASRG is very efficient since there is little consideration of global structure. Our ASRG version conducts local partitioning and tries to form specified regular shapes (e.g., rectangles) for all components if space is available. Space-fill curve techniques adopt space filling strategies but they fill space by forming continuous curves with little consideration of component shapes [19, 45]. Therefore, ASRG may introduce less edge-cut (i.e., communication). Moreover, our GPP is a planar partitioning problem specified for regular meshes, therefore, sphere-cutting approaches are unnecessary and costly [19].

## Algorithms

In this paper, the entire domain is depicted as a *m* by *n* matrix $M\;=\;{\left[s_{i,j}\right]}_{m*n}$. Where *m* and *n* refer to domain size, while an entry *s*_*i*,*j*_ of the matrix denotes an individual subdomain laid on row *i* and column *j* of the domain *M* according to its geospatial locality in grid.

### Objectives & Simplification

Since dust storm simulation targets a specified geographic area with a predefined resolution, the domain size (i.e., granularity of subdomain) is usually fixed or has limited variations. In this paper, we focus on subdomain allocation algorithms. For a given domain size (with known *m* and *n*) and number of computing nodes, an optimal algorithm should be capable of giving solutions to achieve the following objectives:

1. Minimize total (or global) communication cost between computing nodes;
2. Balance workloads of computing nodes;
3. Balance communication among individual computing nodes;
4. Solve the problem efficiently or within acceptable execution time.

For performance and operating purposes, dust storm simulations are usually conducted in homogeneous HPC environments (i.e., computing nodes have the same network condition and computing capability). Therefore, we made simplifications upon a genetic allocation problem, which also makes the algorithms clearly represented. For a concrete domain partitioning, the following heterogeneities are ignored:

1. Heterogeneity on computing capability of computing nodes;
2. Heterogeneity on network condition between computing nodes;
3. Heterogeneity on computing cost for subdomains;
4. Heterogeneity on communication cost for different horizontal neighboring direction;
5. Heterogeneity on computing and communication cost for different vertical geophysical layers (using cube instead of matrix accordingly);

However, the proposed algorithms can be applied to heterogeneous environments by quantifying and normalizing the differences using additive weighting method.

### Integer Linear Programming based Algorithm (ILP)

Linear Programming (LP) is a special type of widely-used mathematical optimization method, which aims to optimize (minimize or maximize) a linear function of variables subject to linear constraints (equality or inequality) [74].

Ideally, the partitioning can be treated as a 0–1 LP problem. For each subdomain s_*i*,*j*_ in domain M, a group of binary variables *x*_*i*,*j*,*k*_ are introduced to describe which component the subdomain belongs to, where 1 ≤ k ≤ c (c is the number of components). If the subdomain s_*i*,*j*_ belongs to the *k*th component, then *x*_*i*,*j*,*k*_ = 1, otherwise *x*_*i*,*j*,*k*_ = 0. A solution of all the n*m*c variables (*x*_*i*,*j*,*k*_) represents a specific partition of the entire domain. Since each subdomain belongs to exactly one component, the following constraint holds $\sum_{k\;=\;1}^{c}x_{i,j,k}\;=\;1$. The number of subdomains in a specific component *k* can be represented as $\sum_{i\;=\;1}^{m}\sum_{j\;=\;1}^{n}x_{i,j,k}$. For two existing neighboring subdomains (*s*_*i*,*j*_ and *s*_*i*′,*j*′_∈{s_*i*,*j*+1_,s_*i*,*j*-1_,s_*i*+1,*j*_,s_*i*-1,*j*_}), if they belong to the same component, then $\sum_{k\;=\;1}^{c}\left|x_{i,j,k}-x_{i',j',k}\right|\;=\;0$, otherwise $\sum_{k\;=\;1}^{c}\left|x_{i,j,k}-x_{i',j',k}\right|\;=\;2$.

Based on this definition, the first three objectives of our algorithm design (section 3.1) can be expressed as Eqs 1, 5 and 2 respectively. The objective function *F* in Eq 1 explicitly describes the first objective (minimizing the total communication). The value of *F* is the total number of edges shared by subdomains separated in different components (total external communication). The number (*P*) of the absolute difference polynomials |*x*_*i*,*j*,*k*_−*x*_i′,j′,k_| in Eq 1 is determined by the total number of neighboring subdomain relations and also the number of components, i.e., *P* = [4m*n−2*m*−2*n*]*c. The second objective (i.e. balancing workload among computing nodes) requires the number of subdomains in each component to be as equal as possible. Therefore, for an ideal partitioning solution with *p* components, the number of subdomains in component *k* should be either ⌊m*n/*c*⌋ or ⌈m*n/*c*⌉(Eq 5). The third objective (i.e., balancing communication among computing nodes) requires that the number of shared edges for each component with other components to be evenly distributed. Eq 2 indicates that the number of shared edges for component *k* must be smaller than *L* (the upper bound of the number of shared edges for component *k*). Theoretically, we can define such a group of inequalities to constrain the number of shared edges for each component. However, for a given number of components (value *c* is flexible), it is tricky to specify a rational *L* for each component. The value of *L* is not only related to the domain size, but also varies with different domain shapes and component locations. Therefore, we ignore this objective.

$$\begin{array}{l} minimize\text{ F}=\frac{1}{4}\sum_{i=1}^{m}\sum_{j=1}^{n}\left(\overset{}{\underset{}{\sum_{k=1}^{c}\left|x_{i,j,k}-x_{i\prime ,j\prime ,k}\right|}}\right) \end{array}$$ (1)

$$\begin{array}{l} \frac{1}{4}\sum_{i=1}^{m}\sum_{j=1}^{n}\left(\left|x_{i,j,k}-x_{i\prime ,j\prime ,k}\right|\right)\leq L \end{array}$$ (2)

Our ILP model treats the objective function as the first objective of algorithm design, while the second objective is treated as a group of constraints. Since Eq 1 consists of absolute difference polynomials, linearization is required. Based on the method of minimizing the sum of absolute deviations [75], a real number difference polynomial *x*_*i*,*j*,*k*_−*x*_i′,j′,k_ is used to represent the difference of two non-negative variables $e_{p}^{+}$ and $e_{p}^{-}$ (i.e.,$\;e_{p}^{+}-e_{p}^{-}$). The corresponding absolute difference polynomial |*x*_*i*,*j*,*k*_−*x*_i′,j′,k_| in the objective function can be substituted by the sum of $e_{p}^{+}$ and $e_{p}^{-}$ (i.e.,$\;e_{p}^{+}+e_{p}^{-}$). In our case, *x*_*i*,*j*,*k*_−*x*_i′,j′,k_ can only be 1, 0 or -1, so we can get the following three states respectively: $e_{p}^{+}\;=\;1,\;e_{p}^{-}\;=\;0$ or $e_{p}^{+}\;=\;0,\;e_{p}^{-}\;=\;0$ or $e_{p}^{+}\;=\;0,\;e_{p}^{-}\;=\;1$. Therefore, the objective function can be linearized by introducing groups of such binary variable pairs. The final ILP model after linearization are defined in Eqs 3 to 7:

$$minimize\text{ F}=\frac{1}{4}\sum_{p=1}^{p}\left(e_{p}^{+}+e_{p}^{-}\right)$$ (3)

subject to the constraints:

$$\sum_{k=1}^{c}x_{i,j,k}=1$$ (4)

$$\sum_{i=1}^{m}\sum_{j=1}^{n}x_{i,j,k}=\left\lfloor \frac{m*n}{c}\right\rfloor \vee \left\lceil \frac{m*n}{c}\right\rceil$$ (5)

$$\left(x_{i,j,k}-x_{i\text{'},j\text{'},k}\right)-\left(e_{p}^{+}-e_{p}^{-}\right)=0$$ (6)

$$x_{i,j,k},x_{i\text{'},j\text{'},k},e_{p}^{+},e_{p}^{-}\in \left\{0,1\right\}$$ (7)

(*i*, *j*, *k*) and (*i*′, *j*′, k) are paired subscript group to ensure that *s*_*i*,*j*_ and *s*_i′,j′_ are neighboring subdomains, while *i* ∈ *N*[1, *m*], *j* ∈ *N*[1, *n*], *k* ∈ *N*[1, *c*]. Each subscript group associates an absolute different polynomial in Eq 1 and a subscript *p* ∈ *N*[1, *P*] for variables $e_{p}^{+}$ and $e_{p}^{-}$. The original objective function (Eq 1) is replaced by a linearized function (Eq 3). Eq 4 indicates that each subdomain belongs to exactly one specific component. Eq 5 means the number of subdomain in each component is fixed and maximally balanced. Eq 6 guarantees the polynomial $e_{p}^{+}+e_{p}^{-}$ can be used to substitute the original absolute difference polynomials |*x*_*i*,*j*,*k*_−*x*_i′,j′,k_|in the objective function. Eq 7 defines the domain of the variables.

### K-Means and Kernighan-Lin Combined Algorithm (K&K)

K-Means can leverage the locality and shapes of components based on the graphical distribution of subdomains in general, but it can neither balance the number of subdomain, nor reduce connections between components. While Kernighan-Lin is a bi-partitioning method which can reduce connections between components effectively, it may get trapped to a local minima [19] and may not produce good results for multiple components partitioning since the algorithm cannot consider partitioning shapes of the entire domain at one time. Therefore, a combination algorithm could leverage advantages and reduce the drawbacks of both methods.

**Fig 2:**
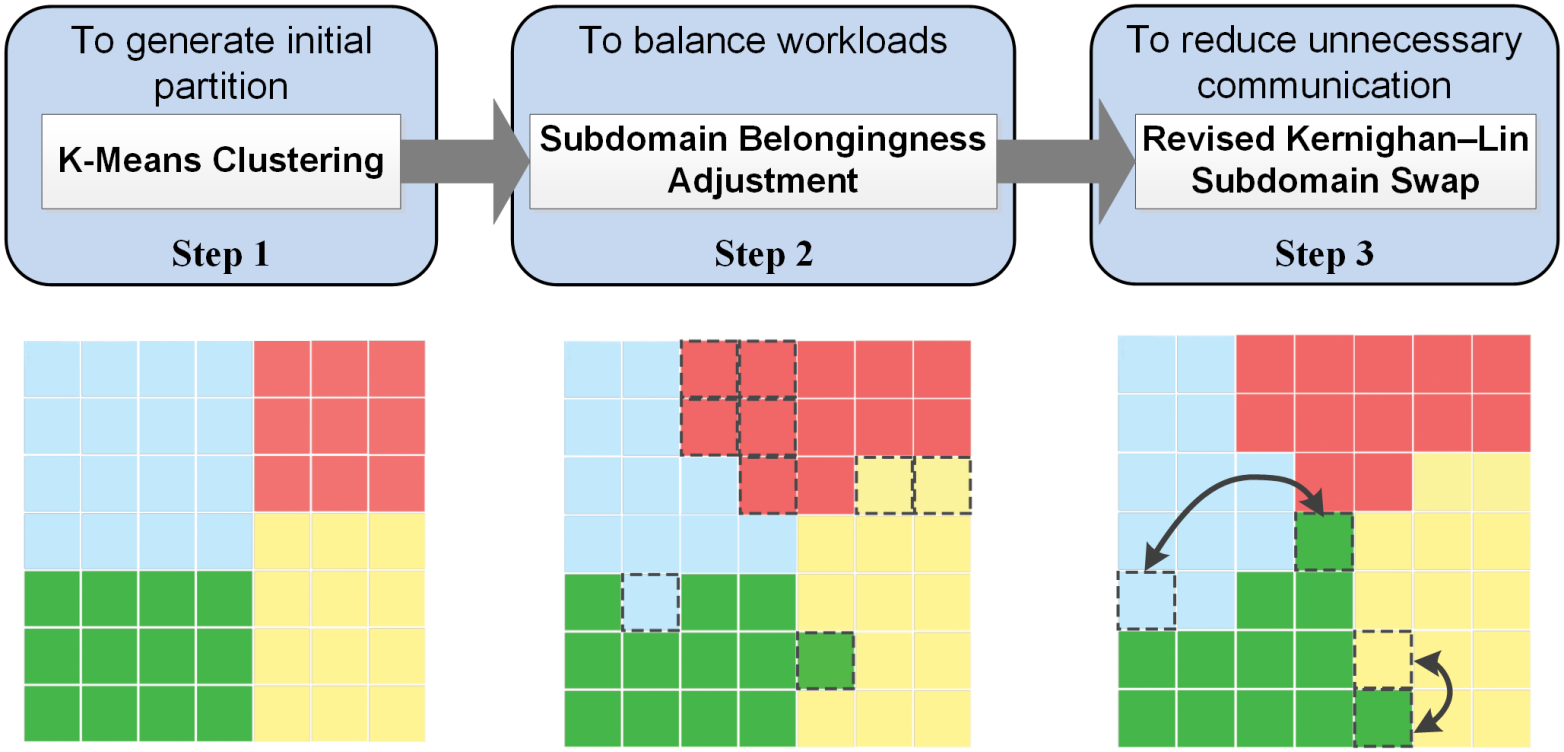
Workflow of K-Means & Kernighan-Lin Combined Algorithm.

The general workflow of the combined algorithm is illustrated in Fig 2 (as an example for partitioning a 7 by 7 domain into 4 components). 1) A K-Means clustering generates the initial partition of subdomains by clustering the subdomains into components (clusters). 2) A subdomain belongingness adjustment changes the belonging of some subdomains after clustering to make the number of subdomains among components to be as equal as possible. 3) A modified Kernighan-Lin algorithm further reduces the number of shared edges by swapping subdomains between neighboring components.

**Fig 3:**
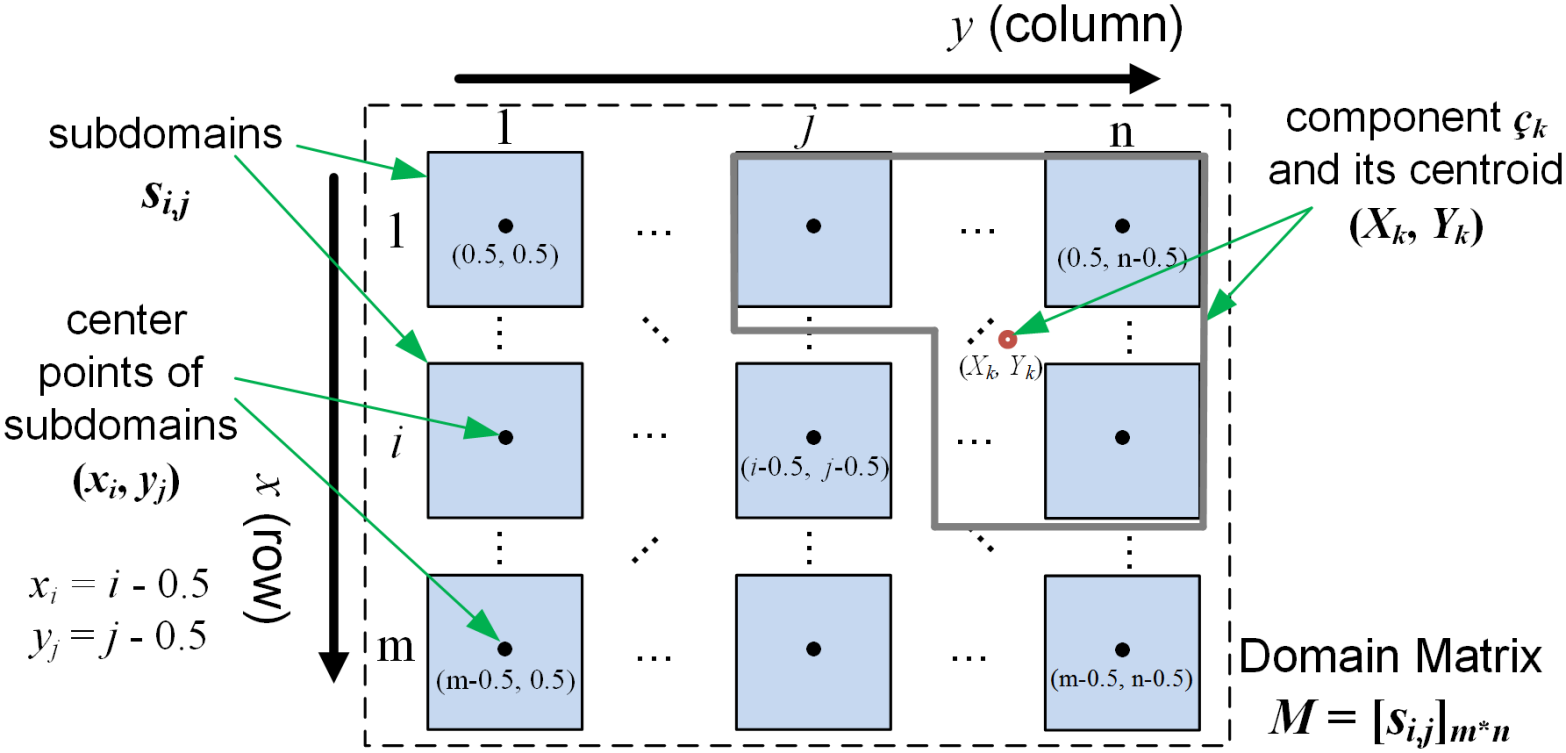
Subdomain Center Points and Component Centroids within a Domain Matrix.

In Step 1, a standard K-Means algorithm is used to calculate the 2-Dimensional (*x*, *y*) squared Euclidean distance between the center point of every subdomain and its component centroid. The algorithm reassigns subdomains to the closest components in an iterative fashion. The center point of a subdomain is defined as illustrated in Fig 3. The centroid of a component is calculated as the mean values of the *x* and *y* coordinates for the center points of all subdomains belonging to the component. The time complexity is O(m*n*c*I*d), where *I* is the number of iterations, d is the dimensions (i.e., 2). Imbalanced subdomain allocation may occur since there is no restriction on the number of subdomain for clusters in a classical K-Means.

**Fig 4:**
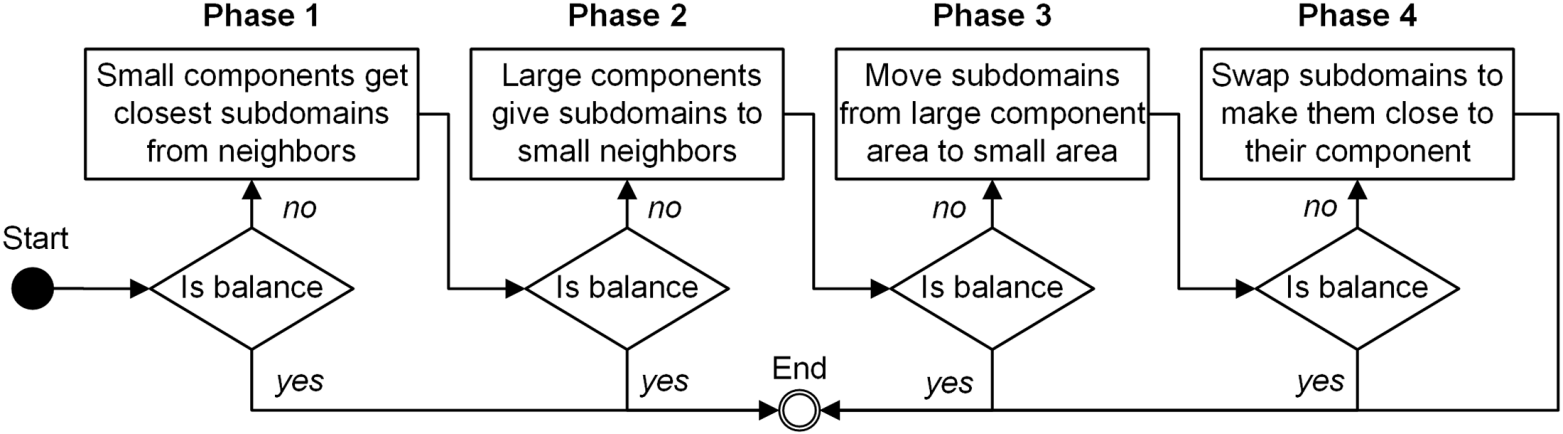
Workflow of Subdomain Belongingness Adjustment.

In Step 2, a four-phase adjustment algorithm (Fig 4) is developed to balance the subdomain number as much as possible. 1) If the number of subdomain in a component is smaller than the average number of subdomains in the neighboring components, then this component will take the closest subdomains from its neighboring components. This process starts from the smallest components in the entire domain. 2) Large components give subdomains to their neighboring small components. 3) Subdomains are moved from the area with large components to the area with small components gradually. 4) Subdomains are swapped to move closer to the center of their component. After each phase, the program will check subdomain numbers. If balanced, Step 2 is completed; otherwise move to the next phase.

**Fig 5:**
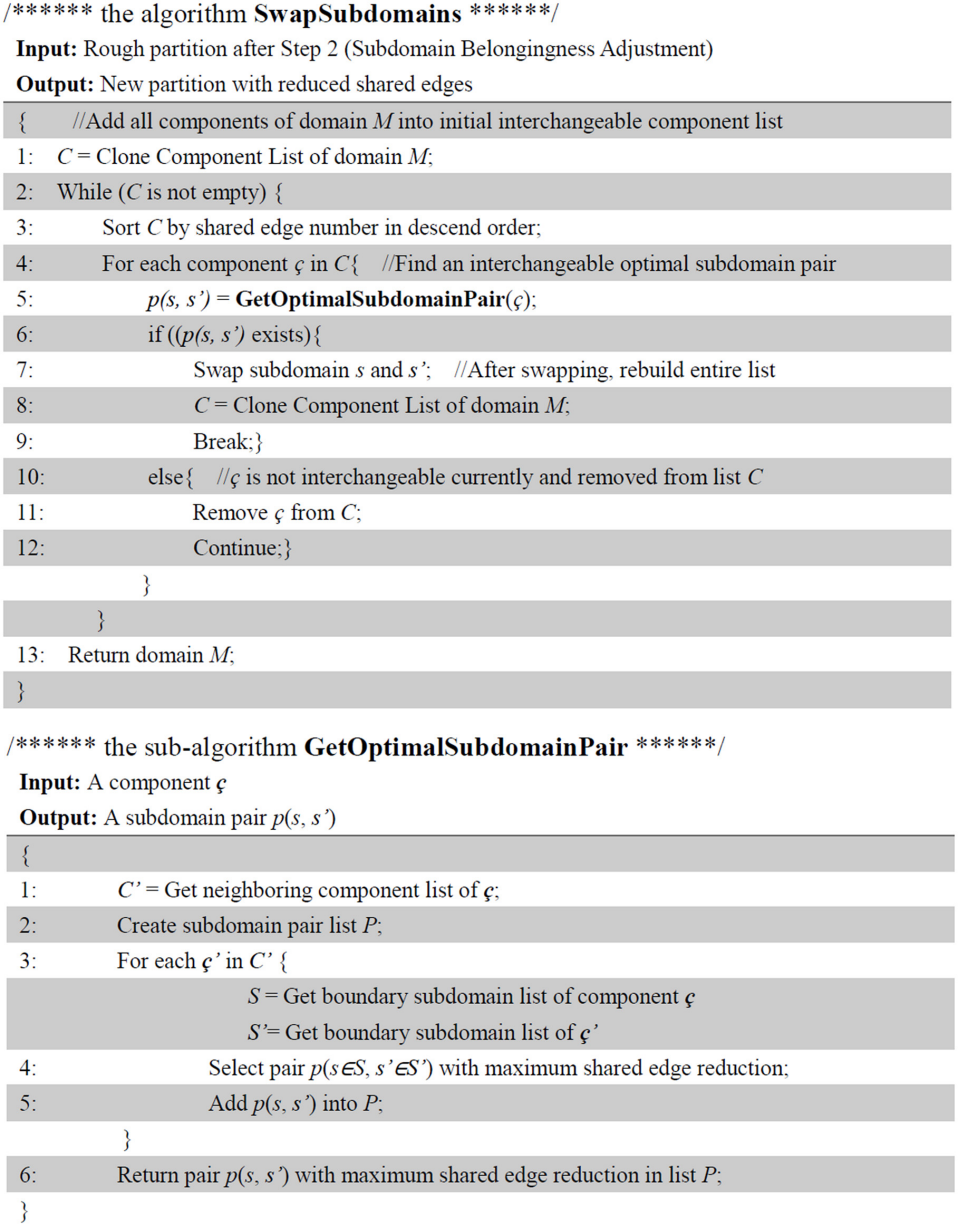
Pseudo codes of Algorithm *SwapSubdomains*.

In Step 3, a modified Kernighan-Lin conducts subdomain swapping for components in pairs. A classical Kernighan-Lin calculates every subdomain pairs (from two different components) to select an optimal series of swapping operation between the two components. Meanwhile, for a domain with *p* components, the total number of component comparison is c*(c-1)/2. Hence, a large domain size and component number will result in high computing cost. Actually subdomain swapping only occurs on the subdomains that locate on the boundary of two neighboring components. Therefore, we simplify the process. The current algorithm only compares two neighboring components and selects subdomains from the boundary of the components. The pseudo codes of the process is described in Fig 5.

### Automatic Seeded Region Growing Algorithm (ASRG)

Seeded Region Growing (SRG) algorithm was invented to partition an image into sub-regions that have similar gray-value pixels according to the locality of pixels [55]. It has been widely used to explore features from images, such as face and fingerprint recognition. This research is to appropriately allocate subdomains of a specific domain matrix into different computing nodes to reduce communications among computing nodes to the full extent. Since the communication only occurs on the neighboring subdomains which belong to different components, neighboring relations need to be considered in the allocation. Thus, all subdomains belonging to the same component are connected is good prerequisite. Such requirement is very similar to SRG image partition. Therefore, SRG algorithm can be also used to detect components in which all subdomains share the same computing node. Based on this idea, an ASRG-based algorithm is implemented and applied to divide computing domain into components. Instead of manual selection of initial seeds for growing in conventional SRG algorithm, the proposed approach enables automatically generating seeds for each component.

**Fig 6:**
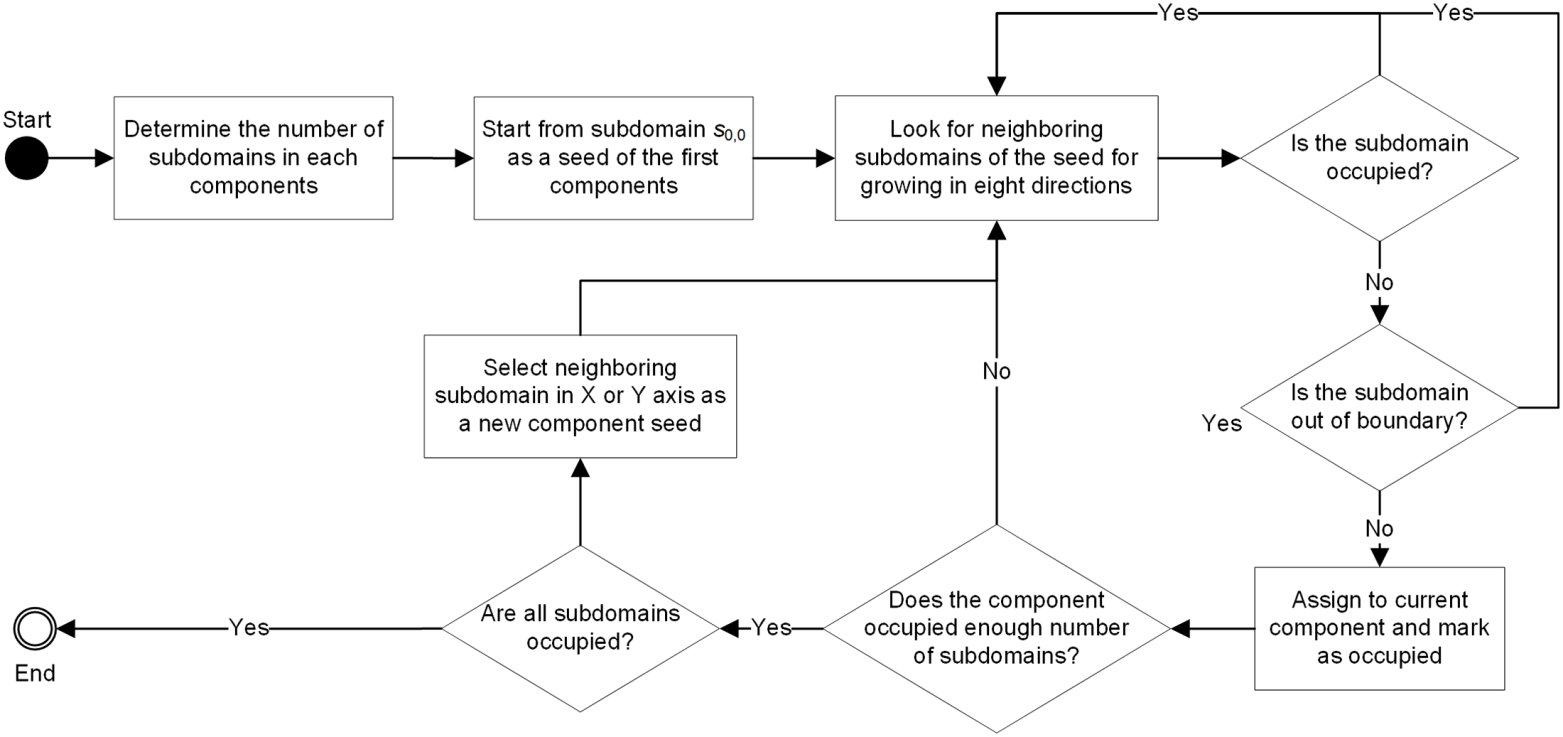
Workflow of ASRG algorithm.

**Fig 7:**
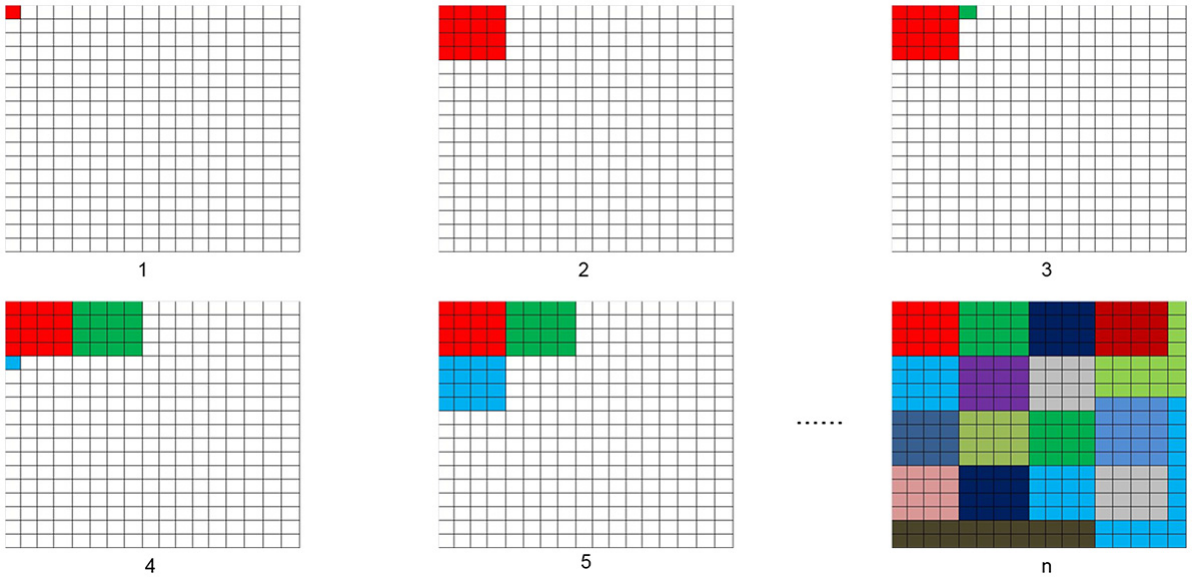
Implementation of ASRG algorithm.

According to the number of components and the size of subdomains, the appropriate number of subdomains with a specific assigned component can be determined by dividing the total number of subdomains by the number of components and rounding it to an integer. The workflow of ASRG algorithm is described in Fig 6. Starting from the first component, the program initializes its original seed at the first entry of the domain (i.e., subdomain *s*_0,0_) and mark it as the current growing seed. Starting from its position, the seed will search through all neighboring subdomains and grow in eight directions. During the searching procedure, current growing seed of a component enables to dynamically change growing directions when coming across the boundary of domain matrix or when the targeting subdomain has been occupied by another component. When a subdomain is occupied, it will be marked as occupied. The process of searching for each component will not stop until the current component is occupied with enough number of subdomains. Once the searching is finished, the program will look for an unoccupied subdomain for initializing seed of the next component by going through the domain. To keep the balance of seed distribution, the program searches for unoccupied subdomains alternatively along X axis and Y axis. Once all subdomains are filled by seeds, the whole process is finished. An exemplificative growing steps of ASRG algorithm are shown in Fig 7.

## Algorithm Comparison Experiment

To assess the three algorithms and investigate the application feasibility, two series of experiments are conducted. In the first series, the algorithms are compared upon their achievements on four objectives defined in section 3.1. The second series of experiments focus on adopting the proposed algorithm on dust storm simulation and investigate the performance improvement compared to default allocation.

### Scenarios Design

The performance and outcome of a partitioning algorithm highly depend on the experimental scenarios. In order to ensure a reliable and comprehensive comparison, the following aspects are considered in the design of experimental scenarios (listed in Table 1): 1) changing domain size; 2) increasing the number of components; 3) different domain shapes (specifically squares, rectangles with different length/width ratios). Due to the computational complexity, we can only test ILP on small domain sizes (i.e., 6 by 6, 10 by 10 and 6 by 20). K&K and ASRG are further test in larger domain sizes, including six squares (20 by 20, 30 by 30, 50 by 50, 60 by 60, 70 by 70 and 100 by 100) and nine rectangles (from 10 by 100 to 90 by 100). To obtain stable result, the average solving time for each algorithm is calculated by repeating five times for each scenario. The number of replicated runs has significant impact on the experiment results and reproducibility to many applications. For example, Arifin et al. [76] demonstrated the importance of the simulation runs to an agent-based model for simulating the larval source of Anopheles gambiae. However, in our case, the partitioning results of the three algorithms keep good immutability and the experimental replication almost has no impact to the partitioning results. In term of solving performance, the differences between the three algorithms are obvious (detailed in section 4.1.3) and few replicated runs is enough to discriminate them. Therefore, we conducted five replications in our experiments to disclose the trends and compare the differences between algorithms. Since the solving time of ILP grows exponentially with both the number of components and the domain size (detailed in section 4.1.3), it may take hours for some experimented scenarios. As a result, partitioning process may become meaningless for real applications due to the time cost. So we force to terminate the solving processes at 600s because K&K and ASRG can get solutions within that time period even for the largest experimental scenario. In that situation, the solutions generated by ILP may not be optimal solutions.

**Table 1:** Scenario Description.

Scenario	Scenario Settings		Tested Algorithm
	Domain size	Component number	
Small Scale (Square)	6 by 6	From 2 to 12	ILP, K&K, ASRG
Small Scale (Square)	10 by 10	From 2 to 25	ILP, K&K, ASRG
Small Scale (Rectangle)	6 by 20	From 2 to 22	ILP, K&K, ASRG
Large Scale (Square)	20 by 20, 30 by 30, 50 by 50, 60 by 60, 70 by 70, 100 by 100	From 2 to 250	K&K, ASRG
Large Scale (Rectangle)	10 by 100, 20 by 100, 30 by 100, 40 by 100, 50 by 100, 60 by 100, 70 by 100, 80 by 100, 90 by 100	From 2 to 250	K&K, ASRG

### Experimental Environment

A desktop with Intel core i7-3770 (3.4 GHz) 8 processors and 16 GB RAM is used as the experimental machine. Operating system is 64-bit Windows 8.0. To compare the performance, we implemented the three algorithms using Java (JDK 7.0). For K&K algorithm, K-Means is implemented by invoking IBM SPSS QUICK CLUSTER function through its Command Line API, while other two steps are coded using java directly. For ILP algorithm, we generate problem description using Java and invoke IBM ILOG CPLEX Optimization studio (http://www.ibm.com/software/products/en/ibmilogcpleoptistud) for solving. The programming language and utilized software/libraries are summarized in Table 2.

**Table 2:** Programming language and used software/libraries for algorithms.

Algorithm	Programming Language & Software & Lib
Integer Linear Programming (ILP)	Java + IBM CPLEX Optimization Studio
K-Means & Kernighan-Lin Combined (K&K)	Java + IBM SPSS
Automatic Seeded Region Growing (ASRG)	Java

### Result Exploration & Analysis

We assess the proposed algorithms based on the objectives of algorithm design described in section 3.1. Due to the limited space, we only use four square domain sizes (6 by 6, 10 by 10, 50 by 50 and 100 by 100) and four rectangle domain sizes (6 by 20, 10 by100, 30 by 100, 60 by 100) to draw the plots. Further quantitative analysis is conducted to make a convincing comparison between K&K and ASRG (described as below) since ILP has unacceptable performance. Ten domain sizes (from 10 by 100 to 100 by 100) with changing width/length rate are selected, which can help to illustrate the potential changing trend of the partitioning result when the domain shape turning from flattened to square. The number of components increases from 2 to 250 on each domain size.

#### Total communication cost

**Fig 8:**
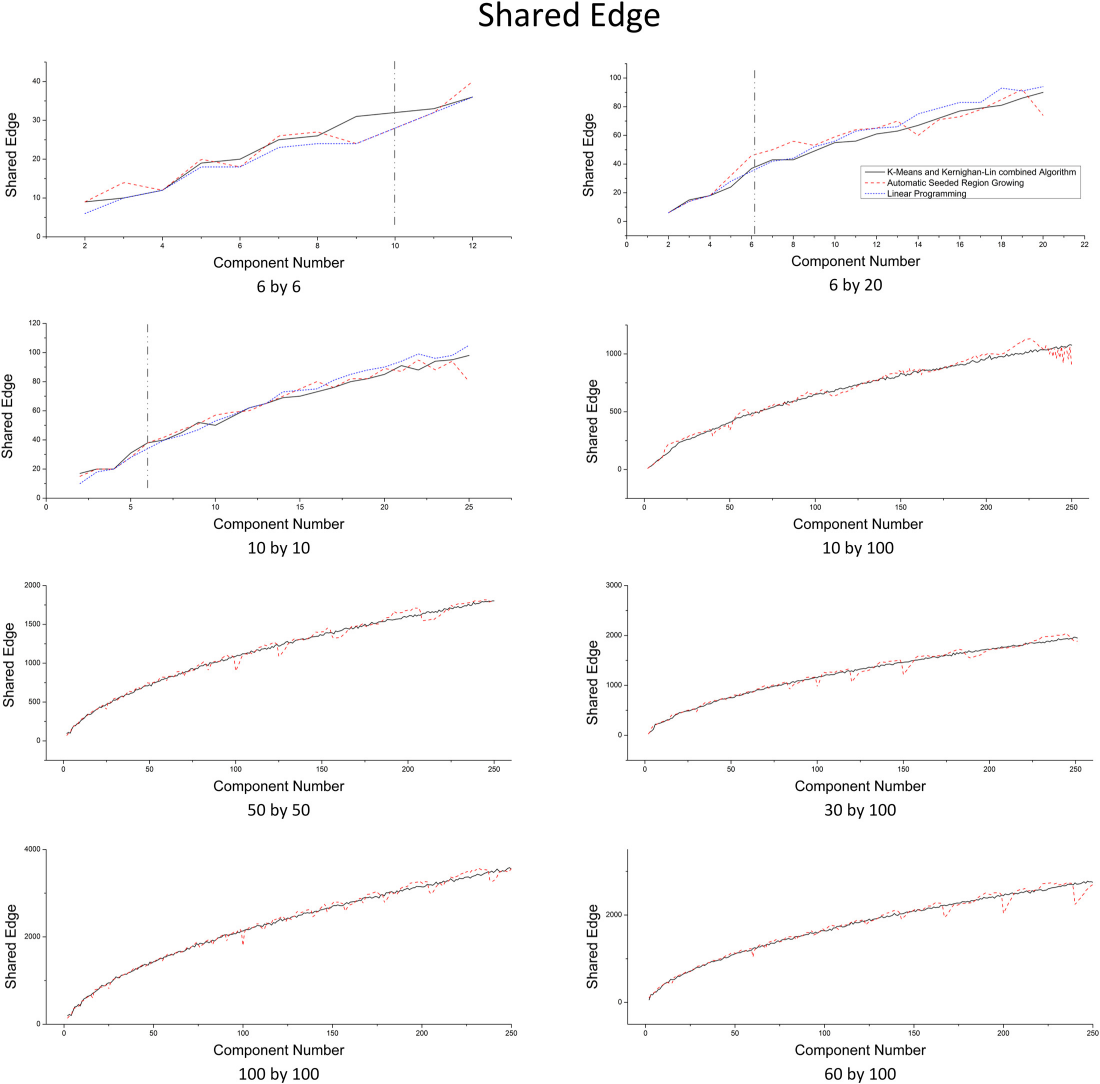
Total Shared Edge Number Comparison for Different Domain Sizes.

The total number of shared edge (TSE) between all component pairs is selected as the measurement of total (global) communication cost between computing nodes (external communication). Theoretically, ILP has the smallest TSE since TSE is modeled as the objective function to minimize. Fig 8 illustrates that in the small scale, ILP gets better result than the other two algorithms when the program ends itself. However, when ILP is forced to terminate due to the extreme computing cost (all cases on the right side of the vertical dash line), the result is even worse than the other two. For specific number of components, ASRG shows obvious drops on TSE (e.g., for 100 by 100 domain size, when the numbers of components are 16, 25 and 100). That is because ASRG can perfectly divide the domain into components with unified regular shapes (e.g., 20 by 20 square) in those situations. To further compare the capability to minimize the global communication quantitatively, we introduce a metric DTSE (Eq 8) which makes the TSE difference between algorithms measurable:

$$D_{TSE}=\frac{TSE_{algorithm1}-TSE_{algorithm2}}{\left(TSE_{algorithm1}+TSE_{algorithm2}\right)/2},$$ (8)

where *TSE*_*algorithm1*_ and *TSE*_*algorithm2*_ are the *TSE* values for the two partitions (with the same domain size and the number of components) generated by algorithm 1 and algorithm 2 respectively. According to the definition, *|D*_*TSE*_*|* can be used to measure the absolute difference (in percentage) of two algorithms. Table 3 illustrates the statistic result on selected domain sizes, where algorithm 1 and algorithm 2 are K&K and ASRG respectively. avg(*D*_*TSE*_) is the mean value of *D*_*TSE*_ for 249 partition pairs (from 2 components to 250 components). avg(|*D*_*TSE*_|) and StD(|*D*_*TSE*_|) are the mean value and the standard deviation of |*D*_*TSE*_| respectively. *D*_*TSE*_(|*D*_*TSE*_| = max) is the *D*_*TSE*_ that its absolutes value |*D*_*TSE*_| is the maximum among the 249 partition pairs and *c*(|*D*_*TSE*_| = max) is the number of components when the maximum |*D*_*TSE*_| occurs. *P*(*D*_*TSE*_<0) is the number of partition pairs in which K&K has smaller *TSE* than ASRG. *Correlation* (*TSE*_*K&K*_, *TSE*_*ASRG*_) is correlation coefficient between the *TSE* value pairs.

**Table 3:** The difference of the total number of shared edges between K&K and ASRG.

	Scenario									
Metric	10 by 100	20 by 100	30 by 100	40 by 100	50 by 100	60 by 100	70 by 100	80 by 100	90 by 100	100 by 100
avg(*D*_*TSE*_)	-0.020769	-0.002388	-0.009327	-0.0082646	-0.0093421	-0.007312	-0.006233	-0.004265	-0.0079132	-0.0007816
avg(|*D*_*TSE*_|)	0.0485845	0.051953	0.0406396	0.0381583	0.034539	0.0377108	0.030602	0.030992	0.0301018	0.0271957
StD(|*D*_*TSE*_|)	0.055789	0.053004	0.052554	0.048984	0.038812	0.050437	0.041852	0.038995	0.035396	0.03217
*D*_*TSE*_(|*D*_*TSE*_| = max)	-0.402204	-0.464646	-0.577075	-0.495726	-0.375479	-0.588235	-0.518519	-0.454106	-0.4	0.324484
*c*(|*D*_*TSE*_| = max)	14	6	4	3	3	2	2	2	2	2
*P*(*D*_*TSE*_<0)	157	141	159	160	161	167	149	149	159	155
*correlation*(*TSE*_*K&K*_, *TSE*_*ASRG*_)	0.991246	0.987698	0.992641	0.991184	0.992463	0.990457	0.995311	0.99577	0.995774	0.99648

From Table 3, we can find that, in general, *TSE* value pairs for K&K and ASRG haves strong positive correlation. The average difference between the two algorithms is trivial according to avg(*D*_*TSE*_), avg(*|D*_*TSE*_*|*) and StD(|*D*_*TSE*_|). But K&K overcome ASRG slightly (avg(*D*_*TSE*_)) and the case that K&K has smaller *TSE* than ASRG is more likely happened according to *P*(*D*_*TSE*_<0). While, there are also some significant differences on *TSE* at extreme cases. Although ASRG has some explicit drops on *TSE* and overwhelms K&K significantly as illustrated in Fig 8, K&K has smaller *TSE* than ASRG when maximum |*D*_*TSE*_| occurs at most cases except 100 by 100 (where K&K partitions the domain along the diagonal, that leads to a conspicuous grows on *TSE*) according to *D*_*TSE*_(|*D*_*TSE*_| = max). Moreover, the largest differences are all happened when the number of components is small (i.e., 2, 3, 4, 6 and 14).

#### Balance of workloads

**Fig 9:**
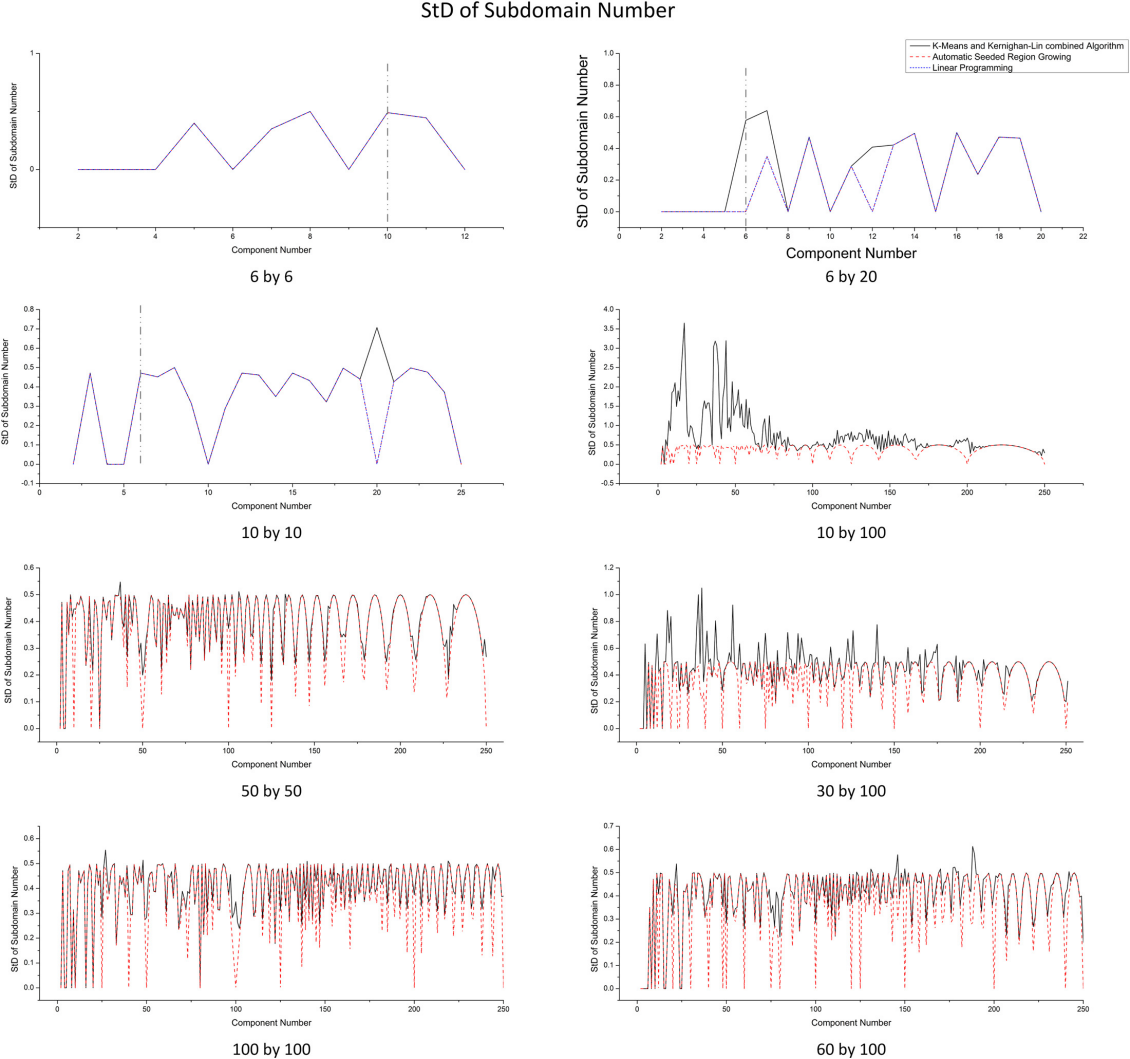
Standard Deviation of the Number of Subdomains in Each Components Comparison for Different Domain Sizes.

The difference on the number of subdomains between components links with the balance of workload (computing cost) directly. The *standard deviation of the number of subdomains* (symbolized as StD(*Sub*), where *Sub* stands for the number of subdomains in a component) of all components in a partition is used as a measurement. A partitioning result with balanced workload has relatively small StD(*Sub*) value. ASRG and ILP have the ability to control *Sub* on each component explicitly, so they generate most balanced solutions (i.e., ideal partitions). K&K needs to adjust *Sub* basing on the initial partition result from K-Means since regular K-Means clustering does not consider the balance of *Sub* among components. The comparison on StD(*Sub*) does not show significant difference among the algorithms in most cases (Fig 9). However, K&K shows unstable results, specifically, when the domain shape is flattened (e.g., 30 by 100 and 10 by 100). This is because K-Means may generate components with extremely imbalanced subdomains, with even empty components. In these extreme cases, the subdomain adjustment operation of K&K algorithm may not eliminate the imbalances completely in current version. To further compare the balance of workload, we defined a new metric *B*_*Sub*_ as Eq 9:

$$\begin{array}{c} B_{Sub}=D_{Sub}-D_{ideal_{Sub}}\\ D_{Sub}=\frac{max(Sub)-min(Sub)}{avg(Sub)}\\ D_{ideal_{Sub}}=\{\begin{array}{cc} 1/avg(Sub)=c/m*n & if\;(m*n)\%c>0\\ 0 & if\;(m*n)\%c=0 \end{array} \end{array},$$ (9)

where max(*Sub*) and min(*Sub*) are the maximum and minimum *Sub* in all components for a partition respectively. avg(*Sub*) are the mean value of *Sub*, which equals to m*n/*c*. So, *D*_*Sub*_ is the maximum difference (in percentage) of *Sub* between any two components in a partition, while *D*_*ideal_Sub*_ is the maximum difference in an ideal partition. The value *D*_*ideal_Sub*_ is 0 if the remainder of m*n/*c* equals to 0; otherwise the value is 1/avg(*Sub*). Therefore, *B*_*Sub*_, the maximum imbalance of *Sub* (in percentage) compared with the ideal situation, can be used to measure the capability to balance the workload of an algorithm. The smaller the *B*_*Sub*_ value is, the more balanced. ASRG and ILP generate ideal partitions, so *B*_*Sub*_ equals to 0. Hence, we analyzed *B*_*Sub*_ for K&K algorithm exclusively. Table 4 is the statistic result for the selected domain sizes. avg(*B*_*Sub*_) is the mean value of *B*_*Sub*_ for 249 partitions in each domain size, while max(*B*_*Sub*_) is the maximum *B*_*Sub*_. *P*(*B*_*Sub*_ *= 0*) is the total number of partitions which has the same value on maximum difference of *Sub* as the ideal partitions.

**Table 4:** The maximum difference on the number of subdomains between K&K and ideal algorithm.

	Scenario									
Metric	10 by 100	20 by 100	30 by 100	40 by 100	50 by 100	60 by 100	70 by 100	80 by 100	90 by 100	100 by 100
avg(*B*_*Sub*_)	0.1119357	0.040169	0.0202276	0.01401	0.011574	0.009407	0.006422	0.006415	0.005794	0.0047414
max(*B*_*Sub*_)	0.5	0.25	0.1666667	0.125	0.08	0.083333	0.071429	0.0625	0.055556	0.05
*P*(*B*_*Sub*_ = 0)	75	111	145	151	156	165	181	170	176	176

From Table 4 and Fig 9, we can find that, although the imbalance may occur, in most cases, the number of subdomains is well balanced. The closer of the domain shape to square, the more balanced. Specifically, avg(*B*_*Sub*_) is larger than 1/10 only in 10 by 100 domain size and the values become less than 1/100 from 60 by 100 domain size.

#### Balance of communications

**Fig 10:**
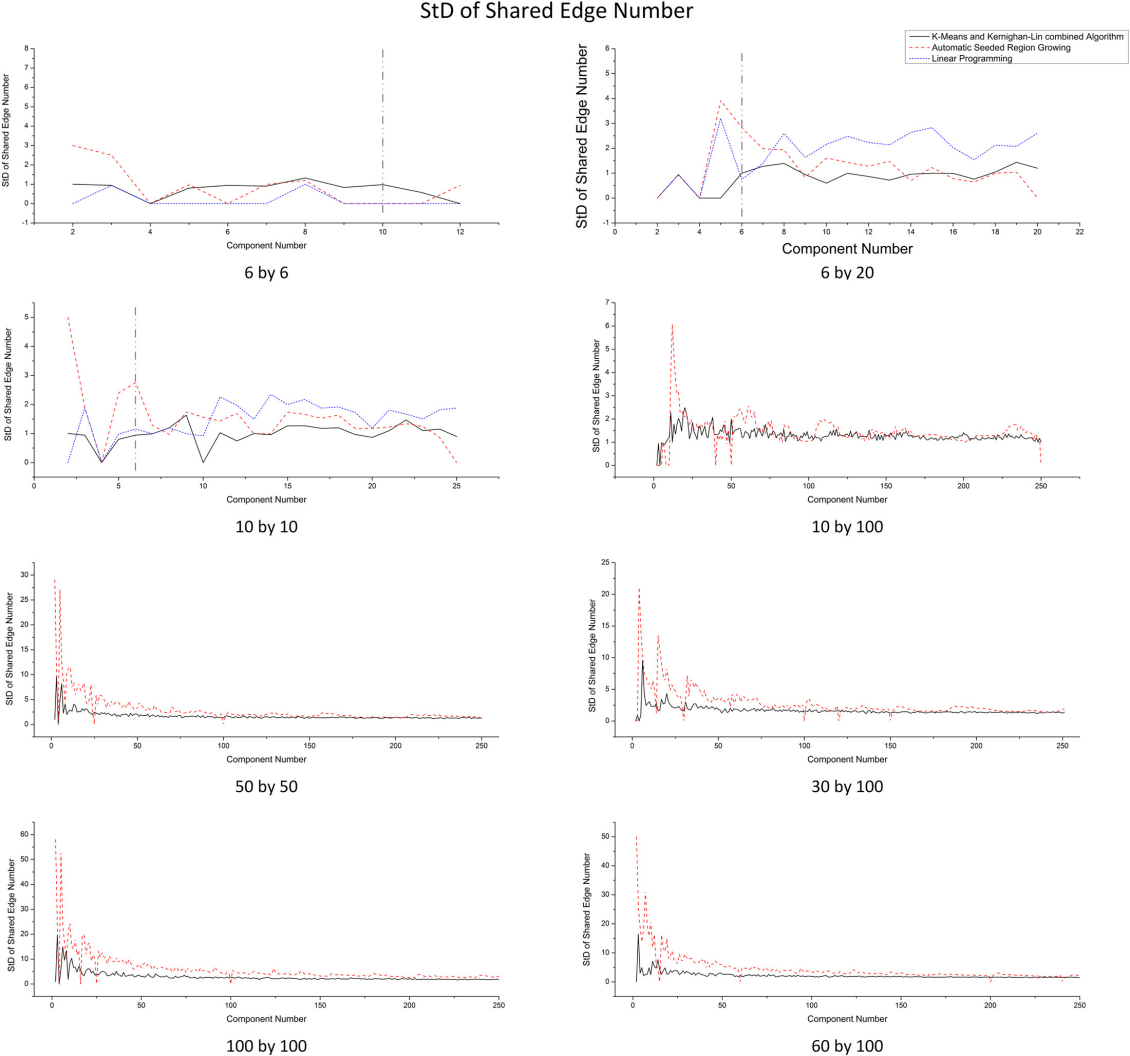
Standard Deviation of the Number of Shared Edges in Each Components Comparison for Different Domain Sizes.

The *standard deviation of the number of shared edges* (symbolized as StD(*SE*), where *SE* stands for the number of shared edges in a component) of all components in a partition is used as a measurement. A partitioning result with balanced communication has relatively small StD(*SE*) value. StD(*SE*) shows that K&K has salient advantage when the number of components is relatively small in general (except 6 by 6), especially when the domain size become larger (shown in Fig 10). The larger StD(*SE*) of ARSG is due to that some components has much more shared edges than the average. ASRG may generate flattened components at the domain boundary or cut a component into isolated parts when there is not enough space left. As the number of components increases, K&K’s advantage becomes less dominant, because for both algorithms, the *Sub* for each component becomes smaller and the *SE*, as well as StD(*SE*), decrease accordingly. To further compare the capability to balance communication cost among components, we define a metrics *D*_*SE*_ as Eq 10:

$$D_{SE}=\frac{\text{max}\left(SE\right)-\text{min}\left(SE\right)}{\text{avg}\left(SE\right)}$$ (10)

*D*_*SE*_ is the maximum difference on *SE* in percentage (the smaller, the better). Where max(*SE*), min(*SE*) and avg(*SE*) are the maximum, minimum and mean value of *SE* for a certain partition respectively. Table 5 is the statistic result on the selected domain sizes. avg(*D*_*SE_K&K*_) and avg(*D*_*SE_ASRG*_) are the mean values of *D*_*SE*_ for 249 partitions in the same domain size for K&K and ASRG respectively, while StD(*D*_*SE_K&K*_) and StD(*D*_*SE_ASRG*_) are the standard deviations. *P*(*D*_*SE_K&K*_<*D*_*SE_ASRG*_ & StD(*SE_K&K*)<StD(*SE_ASRG*)) is the times that both the *D*_*SE*_ and StD(*SE*) of K&K are smaller than the values of ASRG in 249 partitions, i.e., the cases that K&K is more balanced than ASRG on *SE*.

**Table 5:** The maximum difference of the number of shared edges for K&K and ASRG.

	Scenario									
Metric	10 by 100	20 by 100	30 by 100	40 by 100	50 by 100	60 by 100	70 by 100	80 by 100	90 by 100	100 by 100
avg(*D*_*SE_K&K*_)	0.3995752	0.3222584	0.2929574	0.2838793	0.2766204	0.2677715	0.2628887	0.2615701	0.2584333	0.2631178
avg(*D*_*SE_ASRG*_)	0.3763704	0.3881939	0.397379	0.407629	0.4039657	0.4144824	0.4204604	0.4109846	0.4196787	0.4342238
StD(*D*_*SE_K&K*_)	0.1603202	0.1186491	0.0994114	0.0861863	0.0800357	0.08258	0.075161	0.0723531	0.0709068	0.0682374
StD(*D*_*SE_ASRG*_)	0.1421359	0.1269168	0.1187085	0.1210782	0.116409	0.1125606	0.104085	0.1004419	0.0970368	0.1125883
*P*(*D*_*SE_K&K*_<*D*_*SE_ASRG*_ & StD(*SE_K&K*)< StD(*SE_ASRG*))	92	159	192	211	220	223	237	229	236	234

The result implies that K&K is more balanced than ASRG on *SE* on most scenarios except the domain size 10 by 100 (imbalanced *Sub* leads to imbalanced *SE*). Therefore, K&K has an overall better capability for balancing communication cost than ASRG.

#### Algorithm performance

**Fig 11:**
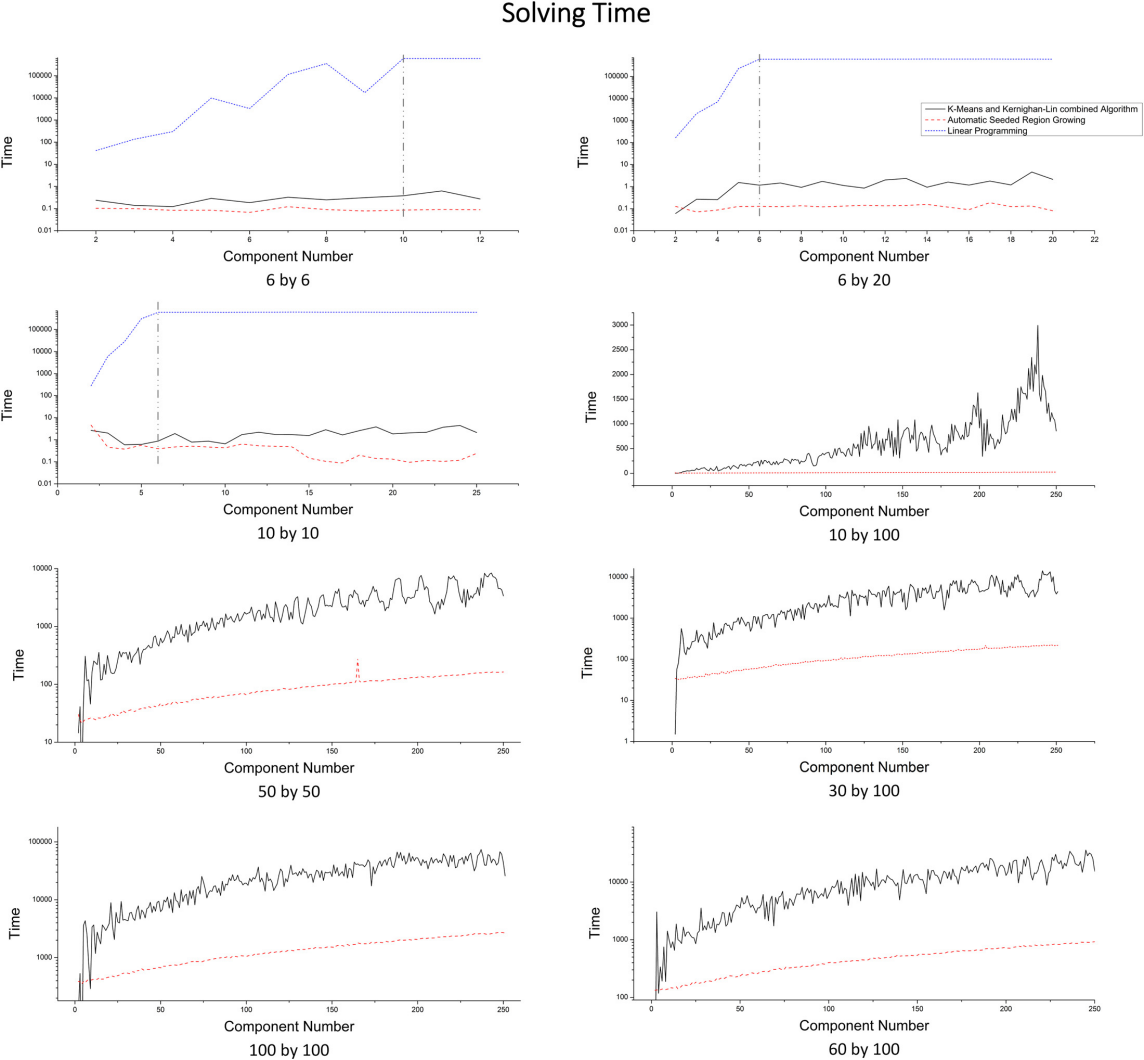
Solving Time Comparison for Different Domain Sizes.

**Fig 12:**
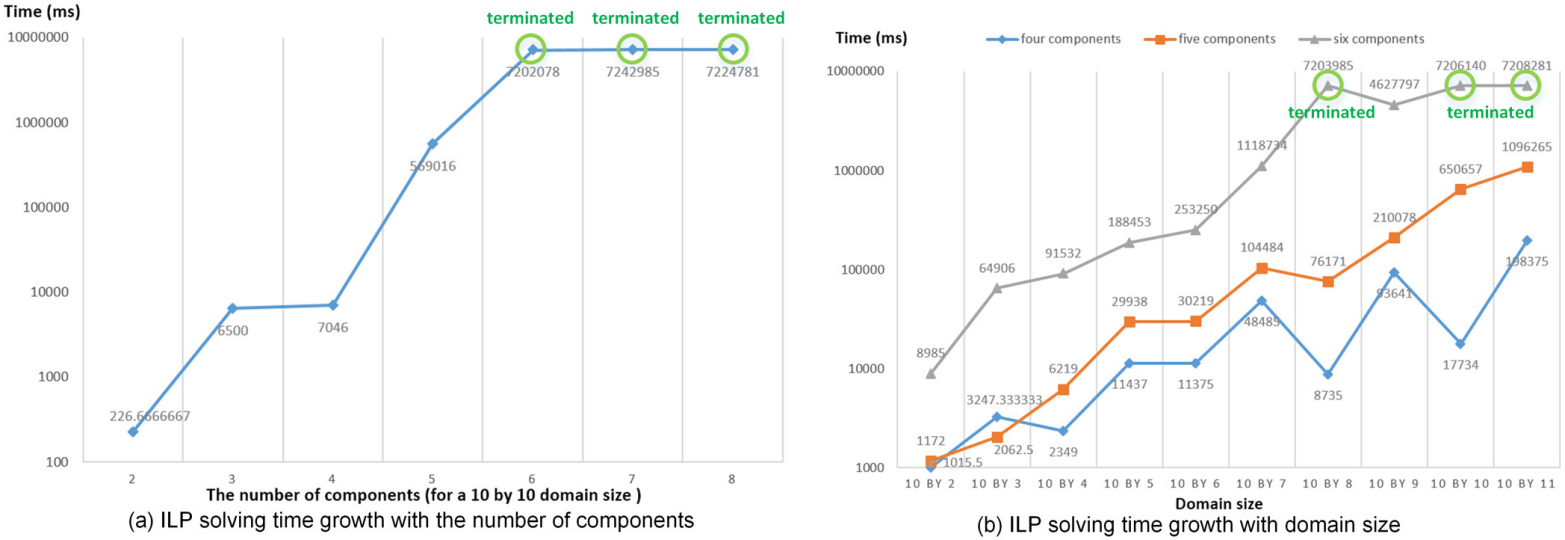
ILP Solving Time Growth with the Number of the Components and the Domain Size (the solving processes were force to be terminated when taking longer than 7200s).

We measure the algorithm performance using the average total time for generating a partition (solving time). Fig 11 shows that ASRG algorithm has the overall shortest time, while ILP has the longest solving time (forced to be terminated at 600s as the vertical dashed line on first three plots) and also the fastest increasing rate. Domain size and the number of components have significant impact on solving time. Fig 12 further illustrates that ILP has exponential growth on solving time with the increasing of the number of components and the domain size. In general, with a fixed domain size, the solving time of the three algorithms increase as component number increases, but there are also some fluctuations. The increasing trend is not obvious on small scale, because the variation of the domain shape and the shape of components have more impact than the domain size and the number of components when the increase on the domain size and the number of components are relatively small. In large scale, relatively, the solving time of ASRG increases stably, while one of the K&Ks has larger fluctuations. That is because when the number of components changes, the balance of *Sub* for the initial partitioning results generated by K-Means varies. In some cases, K-Means generates well-balanced *Sub*; while in other cases, the *Sub* varies a lot. As a result, the time spent on adjusting subdomain belongingness varies a lot and it has large proportion on total solving time.

### Further Discussion

#### Integer linear programming

In this algorithm, the global communication is minimized as the objective function, while workload of each computing node is optimally leveraged by explicitly constraining the number of subdomains in each component. For simplification, the balancing of communication is excluded from the model, but it is partially addressed by the objective function, because the total communication and communication on each component are highly related. In addition, 0–1 LP problem is NP-Hard, and therefore, our algorithm does not scale with domain size and components. In this paper, we experiment with several domain sizes and component by solving a relaxed version of the problem (Eqs 3 to 7).

#### K-Means and Kernighan-Lin combined algorithm

The combined algorithm considers the balancing of both the number of subdomains and the number of shared edges for each component. K-Means ensures that all subdomains belonging to one component are connected. Thus, the communications between components are relatively small in initial partition. Subdomain belongingness adjustment is to make the number of subdomains as balanced as possible. K&K cannot guarantee a perfect balance, especially when the number of subdomains is extremely imbalanced in initial partition. But in general, the results are as good as ideal solutions or have subtle differences in most cases according to Table 4 and Fig 9. Kernighan-Lin minimizes communication between two neighboring components by swapping interchangeable subdomains. Furthermore, K&K can be applied to any irregular domain shapes.

#### ASRG

The distinctive advantages of the ASRG algorithm include low time and space complexity. It is flexible in adjusting and optimizing the seed-growing rules. ASRG can also specify the number of subdomains on each component explicitly. However, it may generate flattened components and even with disconnect subdomains, which will result in relatively high shared edge for these components and imbalanced communication accordingly (Fig 10). Furthermore, if a domain shape is irregular (such as, circle and other polygons), the number of shared edges between components may become difficult to deal with. Therefore, the algorithm is more appropriate for regular domain shapes (e.g., rectangle) with divisible subdomain number.

The comparisons of the three algorithms are summarized in Table 6. The feature *Need Coordinate* shows whether the algorithm requires coordinates for the vertices in partitioning. *Local View* depicts the capability to do localized refinement, while *Global View* measures the capability to consider the entire graph structure in partitioning. *Irregular Domain Shape* measures the support for irregular domain shapes. Others measure the traditional performance from different aspects.

**Table 6:** Comparison of three algorithms through different features.

	Algorithm		
Feature	Integer Linear Programming (ILP)	K-Means and Kernighan-Lin combined Algorithm (K&K)	Automatic Seeded Region Growing (ASRG)
Need Coordinates	No	Yes	Sort of
Local View	Good	Good	Good
Global View	Good	Moderate	Poor
Minimizing Shared Edges	Good / Poor (force terminated)	Moderate	Moderate
Balance of Subdomains	Explicit and Excellent	Moderate	Explicit and Excellent
Balance of Shared Edges	Good / Poor (force terminated)	Good	Poor
Run Time	Slow	Moderate	Quick
Irregular Domain Shape	Good	Good	Poor

## Dust Model Performance Experiment

To make a wise selection from the proposed algorithms for dust storm simulation, the four objectives proposed in section 3.1 are considered. From the performance perspective, ASRG beats K&K and ILP. The computational complexity is the major drawback of ILP in our case, which leads to unacceptable solving performance for real dust storm simulation scenarios. In contrast, the performance difference between K&K and ASRG is trivial comparing to the time saving on dust simulation. For all the dust simulation experiments conducted in this section, the solving time of both K&K and ASRG are less than 0.01s. From the perspective of the workload balance among computing nodes, ILP and ASRG are both efficient. However, the difference of the capability to balance the workload is trivial between K&K and ASRG (Fig 9 and Table 4). The capabilities to minimize the global communication are comparable between K&K and ASRG (Fig 8 and Table 3). Therefore, the capability to balance the communication becomes the major factor, where K&K is superior to ASRG. K&K provides overall most balanced partitioning with acceptable time cost. Furthermore, ASRG cannot handle irregular domain shape in our current version. Complicated rules are needed to be designed to address these problems, while K&K can handle it naturally in general. Therefore, we chose K&K as the allocation algorithm for the dust model experiments. In order to measure the performance impact, we conducted several experiments utilizing K&K to schedule the execution of dust model, and compare the performance results with that from MPI default allocation.

### Scenarios and Experimental Environment

All model performance experimental measurements were obtained in a virtual computing environment managed by Eucalyptus (http://www.eucalyptus.com) in a physical cluster. Up to 8 computing nodes (virtual machine, VM) are used accordingly to experimental scenario and each one composed by 2 CPUs and 2048 MB of memory. In all executions, we simulated dust condition for 3 km resolution, 4.8 by 4.8 degree domain size for 72 hours. The vertical atmosphere layer is divided into 45 layers. Model domain is evenly divided into 4 to 128 subdomains along latitude and longitude directions. For the same computing node number and the same subdomain setting, we ran the model twice, using the default allocation and K&K allocation respectively. Performance is measured by recording the start and end of execution time of each subroutine throughout the model execution.

### Performance Result

**Fig 13:**
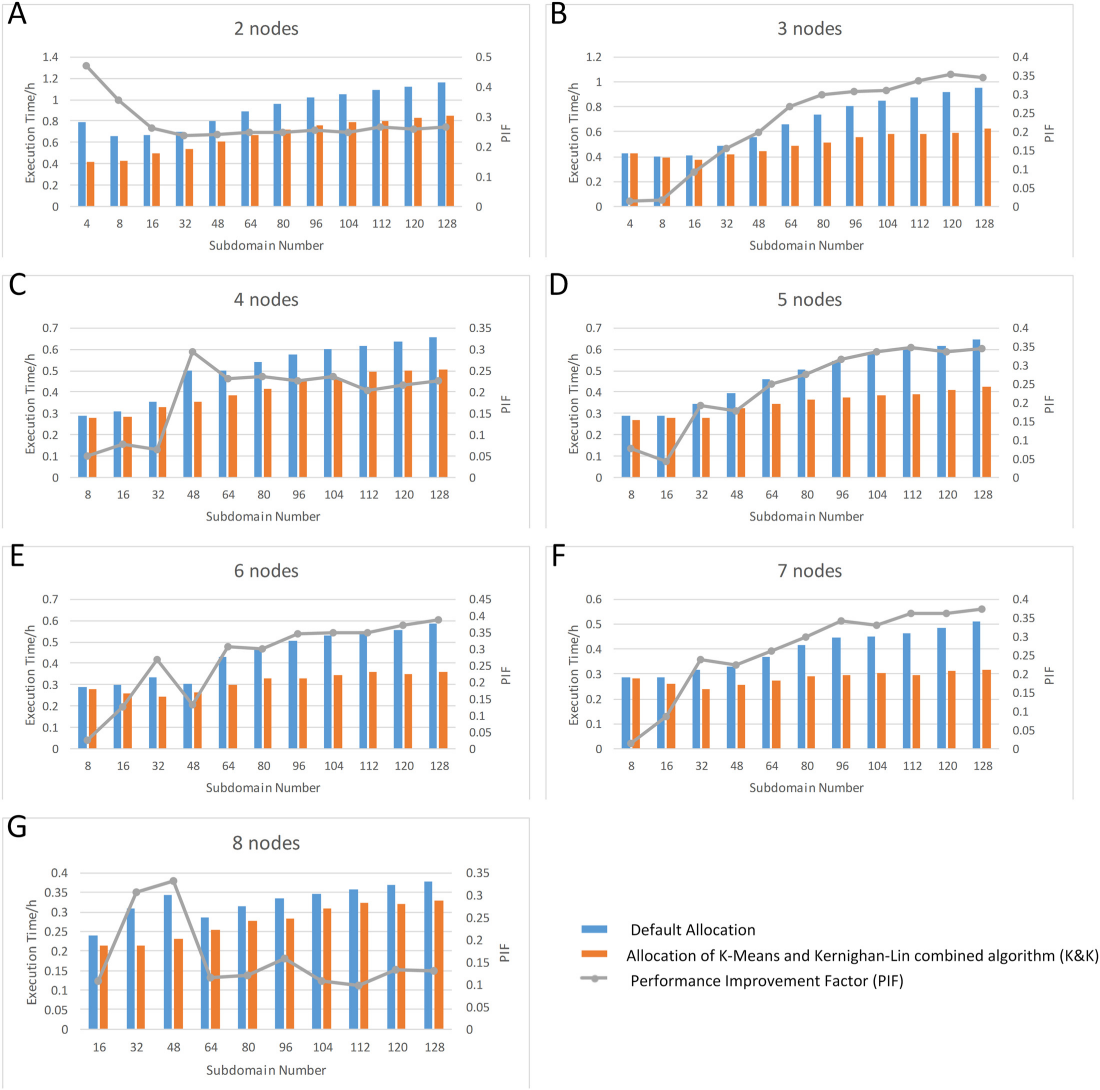
Subdomain Number—Execution Time Plot for Different Node Numbers (Blue bars: execution time using MPI default allocation; Orange bars: execution time using K-Means and Kernighan-Lin combined algorithm (K&K); Grey lines: Performance Improvement Factor (PIF)).

**Fig 14:**
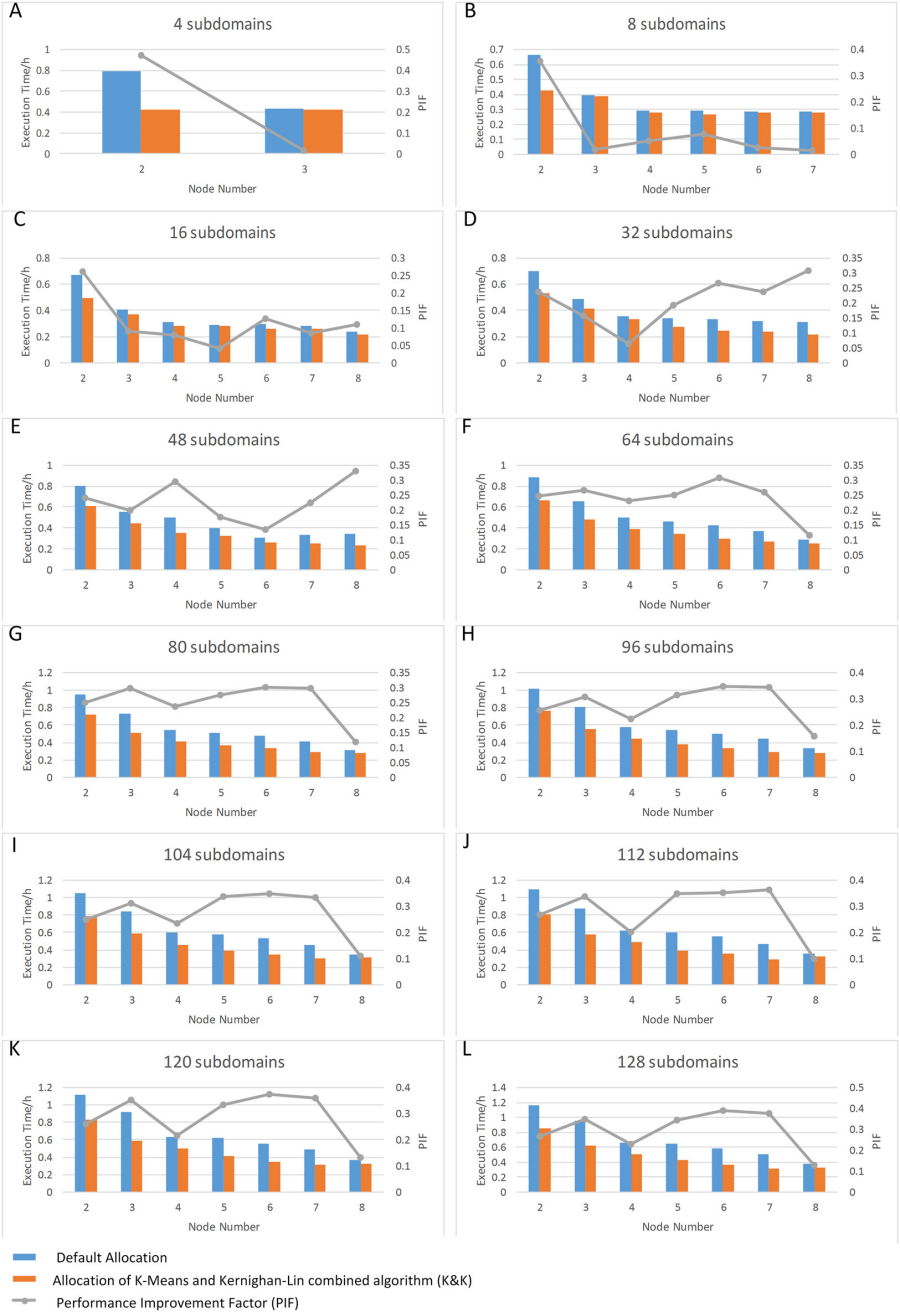
Node Number—Execution Time Plot for Different Subdomain Numbers (Blue bars: execution time using MPI default allocation; Orange bars: execution time using K-Means and Kernighan-Lin combined algorithm (K&K); Grey lines: Performance Improvement Factor (PIF)).

Figs 12–14 show the execution time of NMM-dust model using different computing nodes allocated by K&K and the default allocation method (the non-cluster sequential allocation method implemented by the dust model by default, as illustrated in Fig 1(b)). The overall results support that the proposed method can achieve better performance than default method. Two series of plots, subdomain number vs. time plot and node number vs. time plot, are used to demonstrate the patterns of subdomain number, node number, and execution time (displayed as bar plot). Besides, we illustrate a performance improvement factor on the plots for better demonstration (displayed as grey lines). Here we define performance improvement factor as Eq 11:

$$f=\Delta t/t_{default},$$ (11)

where *t*_*default*_ is default allocation runtime, and Δ*t* is the difference between default allocation runtime and K&K allocation runtime.

### Process Number—Time Plots

Fig 13 illustrates the pattern of the execution time with the number of subdomains varies, using a certain number of computing nodes. It is observed that the overall execution time is increasing, while in some cases execution time is decreasing first and then increasing. The pattern suggests that dividing a domain into finer scale subdomains cannot necessarily reduce execution time, especially when the subdomain number is substantially larger than the node number. There are several possible reasons for that. As the subdomain number increases, the number of shared edges is significantly increased, thus greatly increasing the communication cost, because each shared edge will independently start I/O to conduct data exchange. Since data exchange can exist within the same node, the increased subdomain number also causes local I/O cost to increase.

Fig 13 provides solutions of most suitable subdomain settings for situations when computing resource is limited (fixed maximum number of nodes). Based on the subdomain setting, we can improve model execution efficiency by utilizing the proposed allocation method to allocate those computation tasks.

### Node Number—Time Plots

Fig 14 illustrates the patterns of the execution time with the number of nodes varies, using a certain number of subdomain. As the number of nodes increases for the first several nodes, there is an obvious pattern of decreased execution time. When the increasing node reaches a certain point, the pattern of decreasing execution time turns out to be insignificant. This pattern suggests that we can allocate tasks on relatively low number of computing nodes, but also achieve high efficiency.

### Performance Improvement Factor

**Fig 15:**
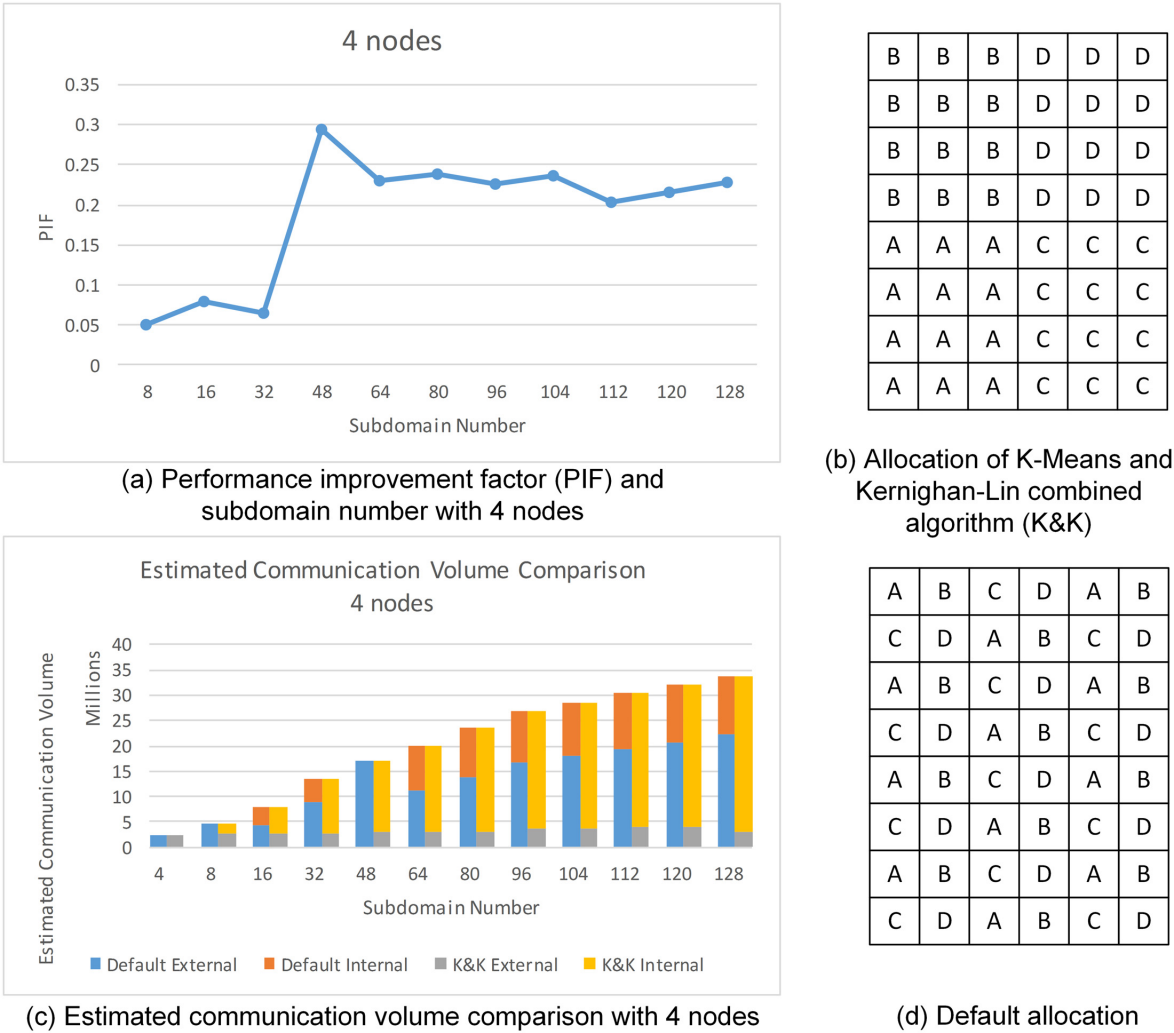
PIF and Estimated Communication Volume for four Nodes. (A) shows the PIF values for 4 nodes. (B) and (D) are the allocation conFig uration using K&K method and default method. (C) is the estimated communication volume (both external and internal) for 4 nodes using two methods. Blue bars: estimated external communication volume using MPI default allocation; Orange bars: estimated internal communication volume using MPI default allocation; Grey bars: estimated external communication volume using K&K allocation; Yellow bars: estimated internal communication volume using K&K allocation.

In both series of plots above, the pattern of performance improvement (displayed in grey lines) varies according to node number and subdomain divisions, where local minima and local maxima may occur at different subdomain number (Fig 13) or node number (Fig 14). Although PIF values indicate that the performance using K&K is overall improved, we can hardly summarize PIF as a simple function of node number or subdomain division. However, the unstable patterns of performance improvement provide the insight that different settings of node number and subdomain divisions generate performance improvements to varying degrees. This phenomenon is derived from the allocation. Take the PIF vs subdomain number plot for 4 nodes (Fig 15A) as an example, there is a noticeable maxima at 48 subdomains. To investigate the reason for this maximum, we compared the results of the two allocation algorithms. It is observed that for the specific situation, K&K generates a regular subdomain division (Fig 15B), while default allocation contains the largest number of shared edges (Fig 15D). Furthermore, Fig 15C illustrates the external and internal communication volume introduced by the two algorithms in each loop. When other conditions remain unchanged, total communication volume is fixed. Therefore, PIF is related to the external and internal communication volume ratio of K&K compared to the one of default allocation. The smaller the ratio of K&K is compared to the default one, the higher is the PIF value, and vice versa.

## Conclusions and Future Work

### Conclusion

To improve the computing performance of subdomain allocation for geoscience and other HPC simulations, this paper introduced and compared three algorithms: 1) an Integer Linear Programming (ILP) based algorithm; 2) a K-Means and Kernighan-Lin combined algorithm (K&K); and 3) an automatic seeded region growing algorithm (ASRG). Four objectives are set to measure the capabilities of algorithms, i.e., minimizing total communication cost, balancing of workload, balancing of communication for computing nodes, and performance. In order to provide a comprehensive assessment of the capabilities and the applicability of these algorithms, we designed two sets of experiments. In the first set, we predefined a number of scenarios for different domain sizes, component numbers and domain shapes. In the second series, we execute the dust storm model allocated by the K&K algorithm, and compared the model performance with the one allocated by the default MPI allocation method.

The first set of experiment result demonstrates that:

1. ILP has the least total communication cost theoretically. It shows best advantage in scenarios when the estimated communication of the program has high cost or the execution environment has limited network bandwidth. The drawback of this algorithm is that when domain size and the number of components increase, the time complexity and space complexity increase exponentially. This characteristic hampers ILP from handling larger scale subdomain allocation problems.
2. K&K provides the most balanced partitioning result in most scenarios. It can best leverage the number of subdomains and the number of shared edges on each component. Furthermore, it has acceptable solving time in large problem scale. K&K can fit into any domain shape, which has great impact on partitioning result.
3. ASRG has the best performance among the three algorithms. It is a good solution for time-critical applications. It can optimize the balance of workload for each computing node, but may introduce imbalances of communications among nodes when there is not enough space left for an expected component shape. However, for some particular domain shapes, ASRG can get optimal partitioning results. In summary, ASRG greatly depends on domain shapes. The locations of initial seeds, growing direction and growing shape are key factors for partitioning irregular domain shapes.

K&K and ASRG have acceptable solving time which makes them applicable to large scale decomposition problems. However, the algorithms have their unique advantages, which can benefit specific application scenarios. These proposed decomposition methods are not only limited to dust storm simulation, but can be applied or extended to support other parallel computing or HPC-based numerical simulation applications that require considering communication cost and computing cost for leveraging multiple computing resources. Moreover, the proposed algorithms are not limited to the simplified scenario described in section 3.1. The generalization methods can be applied to heterogeneous environments, as long as the equilibrium conditions and weights are taken into account.

The second set of experiment result demonstrates that:

1. Dividing a large scale domain into finer scale subdomains cannot necessarily reduce execution time, especially when subdomain number is substantially larger than node number. A good decomposition solution would be giving suggestions about the granularity of subdomain to achieve best resource usage and high efficiency.
2. Total number of computing nodes has a significant impact on execution time. For a certain subdomain settings, allocating tasks on multiple clustered computing nodes can improve execution efficiency to a certain extent.
3. Allocation method is the key factor of accelerating the model execution. External communication has much larger impact on efficiency than internal communication, and whose impact is nontrivial.

### Future Work

1. Investigate the relationship between computing cost and communication cost. To minimize the execution time of numerical simulation, the computing cost and communication cost of each computing node must be best leveraged. The ratios between computing and communication cost on different conditions (e.g., 8-neighors, diversity of neighbors subdomain belongingness) should be investigated. Internal communication will also be considered. With this knowledge, deliberated strategies to refine the allocation may be developed to reassign subdomains from the components with larger shared edges to neighboring components with less shared edges (at the boundaries of the domain).
2. Establish comprehensive decomposition mechanism by integrating multiple approaches. Many algorithms have their own advantage and are suitable for different scenarios. A sophisticated domain decomposition framework can assemble these algorithms and intelligently specify the most suitable algorithm to conduct partitioning according to real application scenarios. To improve the algorithm efficiency, multilevel decomposition strategy should be developed. The strategy divides domain into multiple subdivisions, and conduct decomposition on each subdivision then composite them to a partitioned domain. For particular cases, factorization-based algorithm will also be developed to allocate domain more efficiently and effectively.
3. Consider heterogeneities in decomposition. In order to address more scenarios, it is desired to extend our algorithm and develop a pervasive allocation framework and algorithms. The following heterogeneities should be considered: 1) different computing capabilities and network conditions of computing nodes; 2) communication heterogeneity on both two and three geospatial dimensions (horizontal x, y and vertical z) and computing heterogeneity of different subdomain caused by spatial patterns.
